# Nutritional Supply vs. Flavor Quality: Characterizing the Physicochemical Properties and Amino Acid Profiles of Tomatoes from Beijing and Shandong

**DOI:** 10.3390/foods15101816

**Published:** 2026-05-20

**Authors:** Yiming Zhao, Fengzhi Lyu, Nasi Ai, Qian Yu, Xin Ding, Yanyan Huang, Xiaomin Xu, Ge Chen, Junmei Liu, Donghui Xu, Ming Yang, Guangyang Liu

**Affiliations:** 1 State Key Laboratory of Vegetable Biobreeding, Institute of Vegetables and Flowers, Chinese Academy of Agricultural Sciences, Key Laboratory of Vegetables Quality and Safety Control, Ministry of Agriculture and Rural Affairs of China, Beijing 100081, China; kazuakizhao@163.com (Y.Z.); lyufengzhi@gmail.com (F.L.); gfcyq09@163.com (Q.Y.); dingxin6660906@163.com (X.D.); huangyanyan0412@163.com (Y.H.); xuxiaomin@caas.cn (X.X.); chenge@caas.cn (G.C.); junmei.liu@hotmail.com (J.L.); yangming0422@126.com (M.Y.); 2 National Center of Technology Innovation for Comprehensive Utilization of Saline-Alkali Land, Dongying 257347, China; 3 Changping Field Scientific Observation and Research Station for Agricultural Product Quality and Safety, Ministry of Agriculture and Rural Affairs, Beijing 102202, China; 4 Key Laboratory of Geriatric Nutrition and Health, Ministry of Education, Beijing Technology & Business University, Beijing 100048, China; ainasi@btbu.edu.cn; 5 Gansu Analysis and Research Center, Lanzhou 730030, China

**Keywords:** tomato, nutritional quality, amino acid profile, flavor quality, fruit type, regional differentiation

## Abstract

The nutritional quality and amino acid profiles of 165 tomato samples (66 regular and 99 cherry varieties) from Beijing and Shandong in Northern China were assessed. The results showed that the regional origin was associated with differences in Dry Matter (8.88% vs. 6.73%), Soluble Solids (8.04% vs. 5.80%), total titratable acidity (5.57 vs. 4.08 g/kg), and Lycopene levels (67.32 vs. 38.22 mg/kg). Shandong tomatoes generally showed higher values than those from Beijing. Vitamin C levels were comparable between the two regions (17.79 vs. 13.98 mg/100 g), suggesting no linkage between Vitamin C variation and Dry Matter differences in this dataset. These regional differences likely reflect integrated effects of cultivation systems, varietal composition, and environmental conditions. They may not be explained by geographic origin alone. Principal component analysis revealed regional clustering driven by the accumulation of sugars, organic acids, and amino acids, with glutamate and aspartate contributing strongly to flavor-related variation. These findings provide insights into regional tomato quality and may support precision cultivation and breeding strategies.

## 1. Introduction

Tomato (*Solanum lycopersicum* L.), a member of the genus Solanum within the family Solanaceae, stands as one of the protected vegetable crops with the highest economic value and most extensive cultivation area globally. According to recent statistics from the Food and Agriculture Organization (FAO), the global annual production of tomatoes has surpassed 180 million tons [1], occupying a central position in the international vegetable trade. As the world’s largest producer and consumer, China contributes over 30% of the global total yield, representing a substantial industrial scale.

In China, consumer demand for tomatoes is shifting from quantity-focused supply toward quality enhancement, with particular emphasis on functional nutrition and flavor. However, over the past decades, commercial breeding has largely prioritized yield, disease resistance, and shelf life. This prioritization has been associated with flavor dilution. A retrospective review indicates that traditional breeding and cultivation management have primarily targeted yield, disease resistance, and shelf life/firmness. This selection bias may have reduced alleles associated with flavor and nutrition, a phenomenon often termed flavor dilution [2,3,4]. Consequently, determining how to synergistically improve the intrinsic nutritional quality and sensory flavor of tomatoes through the optimized selection of geographical environments and the rational allocation of varieties, while guaranteeing yield, has become both a focal point and a challenge in interdisciplinary research across horticulture and food science.

As a quintessential representative of functional foods, the tomato is rich in various key bioactive substances, integrating nutritional health benefits with flavor quality [5,6]. Among these, Lycopene serves as the dominant carotenoid in the fruit; as a potent lipid-soluble antioxidant, its capacity to quench singlet oxygen far exceeds that of Vitamin E, holding significant epidemiological importance in reducing the risks of prostate cancer and cardiovascular diseases [7,8,9]. Vitamin C, meanwhile, acts as a crucial water-soluble antioxidant for maintaining cellular redox homeostasis and enhancing immune function. Tomato sensory quality depends on the sugar–acid ratio and on the accumulation of taste-active amino acids such as glutamate and aspartate. These amino acids endow tomatoes with their iconic umami characteristic through synergistic interactions with nucleotides.

The synthesis and accumulation of plant metabolites is a complex dynamic process orchestrated by genotype, environmental factors, and their interaction (G × E), wherein environmental factors play a pivotal role in modifying fruit composition and quality. Specifically, light affects pigment synthesis by regulating the transcriptional levels of key enzyme genes such as PSY1; temperature affects metabolic processes by altering enzymatic kinetic activity [10,11]; and moderate water or salt stress produces a concentration effect by inducing the synthesis of osmolytes such as proline and Soluble Sugar. However, despite advances in understanding single-factor regulatory mechanisms, there is still a lack of systematic comparative data and theoretical analysis of how Shandong (intensive facility production) and Beijing (urban agriculture) shape physicochemical profiles of different tomato fruit types through multifactor coupling [12].

It was hypothesized that Beijing and Shandong, which differ in light, thermal regime, irrigation management, and cultivation technology, would show measurable differences in flavor-related compounds, nutritional components, and amino acid profiles. Nevertheless, varietal composition and agronomic practices inevitably covary with region in field studies and may act as confounding factors; thus, they should be explicitly acknowledged when interpreting regional differences. This study aimed to characterize and compare physicochemical properties, pigment contents, and amino acid profiles of regular and cherry tomatoes from Beijing and Shandong. It also aimed to identify key quality-related compounds and indices associated with regional quality differentiation using PCA, HCA, and Pearson correlation analysis [13]. Finally, it explored compositional associations among sugars, organic acids, pigments, and amino acids under contrasting production environments.

## 2. Materials and Methods

### 2.1. Plant Materials and Pre-Treatments

Shandong Province (34°22′–38°23′ N, 114°47′–122°42′ E) is the largest commercial tomato production base in Northern China, characterized by approximately 2400–2800 annual sunshine hours, a continental monsoon climate with a mean diurnal temperature variation of 10–15 °C during fruit development, and fertile alluvial plains. Commercial tomato production predominantly employs solar greenhouse and plastic tunnel systems, with drip irrigation and integrated water-fertilizer management as standard agronomic practices. In contrast, Beijing (39°26′–41°03′ N, 115°25′–117°30′ E) represents a typical urban facility agriculture zone, with approximately 2000–2400 annual sunshine hours, higher relative humidity, and a greater reliance on overhead irrigation, reflecting a yield- and supply-oriented production strategy.

The samples collected for this study included 44 regular tomato varieties and 39 cherry tomato varieties from Beijing, as well as 22 regular tomato varieties and 60 cherry tomato varieties from Shandong (Table S1). It is acknowledged that the varietal composition of the Beijing and Shandong groups was not fully standardized, as samples were collected from commercial production systems. Therefore, any inter-regional differences observed in this study reflect the integrated effects of environmental conditions, cultivation practices, and varietal composition.

All samples were harvested at the red-ripe stage [14], defined as Color Stage 6 (>90% red surface coverage per the USDA chart) and meeting minimum TSS thresholds. Regular tomatoes required ≥4.0 °Brix, while cherry tomatoes required ≥6.0 °Brix. All harvesting decisions were made by the same trained personnel. Following harvest, samples were stored under refrigeration (4 °C) and analyzed within 48 h. Prior to analysis, fresh tomato samples (approximately 200 g per sample, consisting of 5–10 representative fruits) were homogenized into a uniform pulp using a laboratory blender at high speed (~10,000 rpm) for 60 s. The homogenized samples were immediately subjected to measurement to prevent metabolite degradation.

### 2.2. Reagents and Chemicals

The reagents used in this study included ethanol (HPLC grade, Avantor, Radnor, PA, USA); methanol (HPLC grade, Avantor); n-hexane (HPLC grade, Avantor); chloroform (analytical grade, Avantor); 2,6-dichlorophenolindophenol solution (analytical grade, Chemicell, Berlin, Germany); Lycopene standard (≥95% purity, Macklin, Shanghai, China); anhydrous sodium chloride (analytical grade, Macklin); anhydrous sodium sulfate (analytical grade, Aladdin); sodium hydroxide solution; phenolphthalein indicator (analytical grade, Macklin); tartaric acid (analytical grade, Aladdin); glucose standard (≥99.5% purity, Rhawn, Shanghai, China); Vitamin C standard (L-ascorbic acid, ≥99% purity, Pureone, Mumbai, India); sodium acetate (analytical grade, Aladdin, Shanghai, China); concentrated sulfuric acid (analytical grade, Nanjing Reagent, Nanjing, China); copper sulfate (analytical grade, Aladdin); hydrochloric acid standard solution (analytical grade, Nanjing Reagent); acetone (analytical grade, Avantor); acetonitrile (HPLC grade, Nanjing Reagent); amino acid mixed standards (Sigma-Aldrich, St. Louis, MO, USA); and derivatization reagents (Sigma-Aldrich). Distilled water and deionized water were used throughout. All other reagents were of analytical grade unless otherwise specified.

### 2.3. Determination Methods

Lycopene Determination: Lycopene is a highly hydrophobic carotenoid, and it is prone to oxidative degradation during extraction and sample handling, as well as structural disruption caused by light. Following GB/T 22249-2008 [15], a phloroglucinol–dichloromethane system was used as the main extraction solvent. Dichloromethane can dissolve lycopene more effectively, thereby improving the extraction efficiency of hydrophobic components, while phloroglucinol provides antioxidant activity to suppress oxidation during extraction, improving analyte stability and the reliability of the determination.

To minimize the effects of oxidation and light exposure, sample pretreatment and solution preparation were carried out under light-protected conditions. Specifically, weighing, extraction, and volume adjustment were performed in amber volumetric flasks. After filtration (0.45 μm), the extract was stored in a light-protected environment. The lycopene reference standard was stored under light protection and at low temperature according to the standard procedure, and the standard solutions were prepared fresh prior to use. In addition, the time from completion of extraction to injection was kept as short as possible to reduce the risk of degradation.

For sample preparation, 0.5–2.0 g of homogeneous sample (precision 0.001 g) was weighed into a 25 mL amber volumetric flask, and 20 mL of the phloroglucinol–dichloromethane solution was added for light-protected ultrasonic extraction for 30 min. The solution was then brought to volume with the same solvent, mixed thoroughly, and filtered to obtain the sample solution. The calibration curve was prepared using this solvent system. The standard concentrations were 0.2, 0.5, 1.0, 5.0, 10.0, and 20.0 μg/mL. Identification was based on retention time, while quantification was performed by establishing a linear relationship between peak area (or peak height) and concentration. The linear range was 0.200–20.0 μg/mL. The HPLC conditions included a Waters Acquity UPLC BEH C18 column (100 mm × 2.1 mm, 1.7 μm), column temperature of 30 °C, detection wavelength of 472 nm, mobile phase: methanol + acetonitrile = 50 + 50 (*v*/*v*), flow rate of 1.0 mL/min, and injection volume of 10 μL.

Carotene Determination: Carotenoids were determined according to GB 5009.83-2016 [16]. Before extraction, saponification was carried out on liposoluble carotenoids to release the pigments bound to the matrix. After pretreatment, the sample solution was added to anhydrous ethanol and a potassium hydroxide solution and then was placed in a thermostatic shaking water bath at 53 ± 2 °C for 30 min; if the saponification was not complete, the reaction time could be appropriately extended to 1 h. After cooling to room temperature, the subsequent petroleum ether extraction was performed, and the obtained solution was dissolved in dichloromethane, made up to volume, and filtered, yielding the test solution for analysis.

The analytical column was a C18 column (250 mm × 4.6 mm, 5 μm), or an equivalent column. The mobile phase consisted of chloroform–acetonitrile–methanol (3:12:85, *v*/*v*/*v*) containing 0.4 g/L ascorbic acid and was filtered through a 0.45 μm membrane before use. The flow rate was 2.0 mL/min, the detection wavelength was 450 nm, the column temperature was maintained at 35 ± 1 °C, and the injection volume was 20 μL.

Quantification was performed using the external standard method to establish a calibration curve. The β-carotene standard working solutions were prepared according to the standard; the calibration concentrations were 0.5, 1.0, 2.0, 3.0, 4.0, and 10.0 μg/mL. Linear regression was carried out using peak area versus concentration, giving the regression equation y = 87,543x − 6039, R^2^ = 0.9993. The linear range of the calibration curve in this study was 0.5 μg/mL to 10 μg/mL, and sample quantification was performed within this range. The absolute difference between the results of two independent determinations did not exceed 10% of the arithmetic mean. The limit of detection (LOD) and limit of quantification (LOQ) were calculated based on a sample mass of 5 g: LOD = 0.5 μg/100 g and LOQ = 1.5 μg/100 g. No internal standard was used in this study.

Amino Acid Determination: Amino acids were determined using an automatic amino acid analyzer (Sykam S–4331) equipped with ninhydrin post-column derivatization and ion-exchange chromatography, in accordance with GB 5009.124-2016 [17]. The homogenized sample was accurately weighed into a pressure-resistant hydrolysis tube so that the protein content was generally within 10–20 mg. Then, 10–15 mL of 6 mol/L HCl was added according to the protein content of the sample, followed by 3–4 drops of phenol. The hydrolysis tube was placed in a freezing bath for 3–5 min, connected to a vacuum pump, evacuated to nearly 0 Pa, and then filled with nitrogen. The vacuum–nitrogen cycle was repeated three times, and the tube was sealed under nitrogen.

Hydrolysis was performed at 110 ± 1 °C for 22 h in an electric constant-temperature oven or hydrolysis furnace. After cooling to room temperature, the hydrolysate was filtered into a 50 mL volumetric flask. The hydrolysis tube was rinsed several times with a small amount of water, and the rinsing solutions were combined in the same volumetric flask. The solution was then diluted to volume with water and thoroughly mixed. An aliquot of 1.0 mL of the filtrate was transferred into a 15 mL or 25 mL tube and evaporated to dryness under reduced pressure at 40–50 °C using a tube concentrator or parallel evaporator. The residue was dissolved in 1–2 mL of water and evaporated again under reduced pressure to remove residual acid and finally dried completely. The dried residue was reconstituted with 1.0–2.0 mL of pH 2.2 sodium citrate buffer, mixed thoroughly, filtered through a 0.22 μm membrane, and transferred to an autosampler vial for analysis.

The mixed amino acid standard stock solution was prepared at 1 μmol/mL, and the mixed amino acid working solution was prepared at 100 nmol/mL by dilution with pH 2.2 sodium citrate buffer. The standard working solution and sample solution were injected into the amino acid analyzer at the same injection volume. Amino acid concentrations in the sample solution were calculated by the external standard method based on peak areas.

The chromatographic separation was performed on a sulfonic acid-type cation-exchange resin column. The derivatized amino acids were detected at 570 nm and 440 nm. The elution buffers, ninhydrin reagent, operating program, and instrumental parameters were prepared or adjusted according to the instrument manual and GB 5009.124-2016 to ensure stable operation.

Other Physicochemical Indexes: Dry Matter, Total Acid, Soluble Sugar, total Soluble Solids (TSS), Vitamin C, and Protein were determined in accordance with the relevant National Standards of the People’s Republic of China (GB).

### 2.4. Data Processing and Statistical Analysis

All measurements were performed in triplicate. Data compilation was conducted using Microsoft Excel 2021. Statistical analyses including Pearson correlation analysis, Principal Component Analysis (PCA), and Hierarchical Cluster Analysis (HCA) were performed using Origin 2022 software to evaluate the inter-varietal differences and correlations among Lycopene, Dry Matter, Total Acid, Soluble Sugar, TSS, Vitamin C, Protein, carotene, and 16 amino acids.

#### 2.4.1. Descriptive Statistics and Significance Testing

Descriptive statistics (mean ± standard deviation, SD) were calculated for all quality parameters [18]. Independent-samples *t*-tests were used to compare quality indices between Beijing and Shandong groups, performed separately for regular tomato groups and cherry tomato groups. Statistical significance was set at *p* < 0.05, with *p* < 0.01 and *p* < 0.001 indicating highly significant differences.

#### 2.4.2. Pearson Correlation Analysis

Pearson correlation coefficients (r) were calculated to quantify the linear association between quality parameters [19]. Significance was tested using a two-tailed *t*-test.

#### 2.4.3. Principal Component Analysis (PCA)

PCA with Z-score normalization was employed to reduce data dimensionality and visualize sample clustering patterns and variable contributions. Biplots displaying sample scores and variable loadings were generated for interpretation [20].

#### 2.4.4. Hierarchical Cluster Analysis (HCA)

HCA was performed using Euclidean distance and Ward’s minimum variance method [21]. Prior to HCA, amino acid data were autoscaled (mean-centered and divided by the standard deviation of each variable). Results were visualized as dendrograms and clustered heatmaps.

#### 2.4.5. Data Visualization

Box plots were generated to display the distribution characteristics of quality indices, including median, interquartile range, and potential outliers. Correlation heatmaps were constructed to visualize the magnitude and direction of pairwise correlations among quality parameters, with color intensity representing correlation strength.

## 3. Results

### 3.1. Analysis of Flavor and Texture Quality Differences in Regular Tomatoes from Different Origins

**Figure 1:**
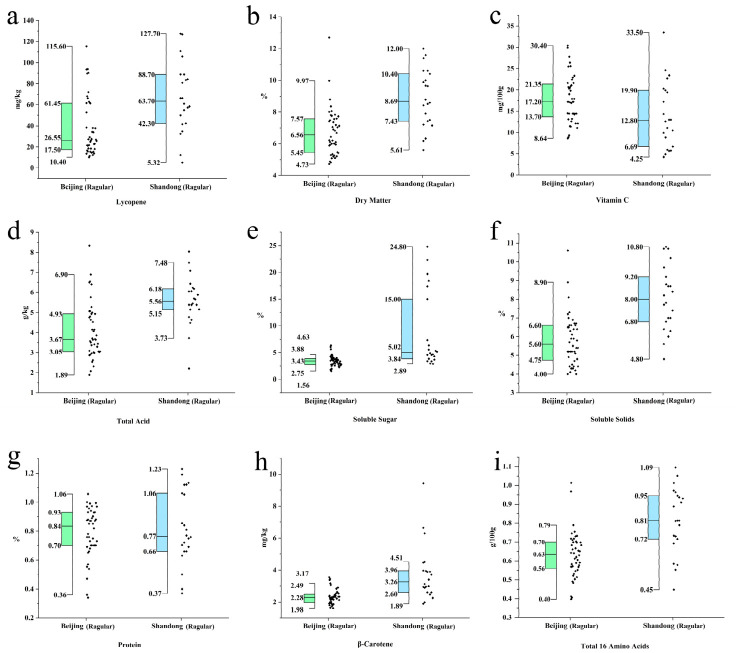
Comparison of physicochemical properties and nutritional components between regular tomatoes from Beijing and Shandong. (**a**) Lycopene; (**b**) Dry Matter; (**c**) Vitamin C; (**d**) Total Acid; (**e**) Soluble Sugar; (**f**) Soluble Solids; (**g**) Protein; (**h**) β-Carotene; (**i**) Total 16 Amino Acids. In the boxplots, the horizontal line within the box represents the median, the upper and lower edges of the box represent the upper and lower quartiles, and the whiskers denote the upper and lower edges of the data. The corresponding mean values are presented in Table 1.

Flavor and texture are the core factors determining the eating quality and consumer acceptance of tomatoes. A comparative analysis was conducted on sugar-acid and texture-related indices for samples from Beijing and Shandong (Table 1, Figure 1).

It should be noted that the varietal composition of the two regional groups was not fully standardized; therefore, the following regional differences reflect the integrated outcome of environmental conditions, cultivation practices, and varietal composition, rather than the effect of geographical location alone. Independent samples *t*-test results demonstrated that samples from the Shandong region were significantly superior to those from Beijing in key indicators determining flavor intensity. Specifically, as shown in Figure 1, the mean Soluble Solids content in the Shandong group was 8.04%, which was significantly higher than the 5.80% observed in the Beijing group (*p* < 0.001). Similarly, the mean Soluble Sugar content reached 8.66%, significantly higher than the 3.41% in the Beijing group (*p* = 0.0025). Regarding acidity, the mean Total Acid content in the Shandong group (5.57 g/kg) was also significantly higher than that in the Beijing group (4.08 g/kg, *p* < 0.001). Both sugar and acid levels were simultaneously higher in the Shandong group [22].

Furthermore, the analysis of the total content of 16 amino acids (Figure 1f) showed that the Shandong group (0.82 g/100 g) was significantly higher than the Beijing group (0.63 g/100 g).

In terms of texture, the Dry Matter content (Figure 1a) in the Shandong group (8.88%) was highly significantly higher than that in the Beijing group (6.73%, *p* < 0.001).

#### 3.1.1. Comparison of Functional Pigments and Antioxidant Components

Lycopene and β-Carotene are not only the primary pigments determining tomato fruit color but also vital antioxidants for human health [23,24]. The analysis results (Figure 1b,i) indicated that samples from the Shandong region possessed a significant advantage in the accumulation of lipid-soluble pigments.

The mean Lycopene content in the Shandong group reached as high as 67.32 mg/kg, significantly exceeding the 38.22 mg/kg found in the Beijing group (*p* = 0.0012). Similarly, the β-Carotene content in the Shandong group (3.76 mg/kg) was significantly higher than that in the Beijing group (2.32 mg/kg).

#### 3.1.2. Analysis of Basic Nutritional Components

In contrast to the substantial differences observed in flavor and pigment indices, the variation in basic nutritional components between the two groups was relatively minor. Protein content (Figure 1c) showed no statistically significant difference between the two groups (*p* = 0.613), with mean values of 0.79 and 0.82 for the Beijing and Shandong groups, respectively. Regarding Vitamin C (Vc) content (Figure 1h), the Beijing group mean (17.79 mg/100 g) was slightly higher than the Shandong group (13.98 mg/100 g), showing a significant difference (*p* = 0.048). Notably, the Vc difference was marginal (*p* = 0.048), and the effect size was smaller than that observed for sugar and acid indices.

### 3.2. Difference Analysis of Quality Indices of Cherry Tomatoes in Beijing and Shandong Regions

**Figure 2:**
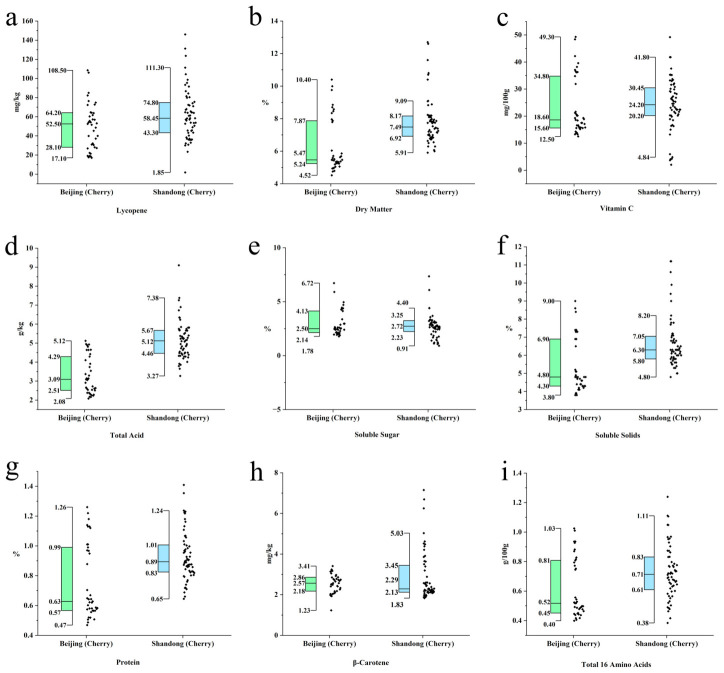
Comparison of physicochemical properties and nutritional components between cherry tomatoes from Beijing and Shandong. (**a**) Lycopene; (**b**) Dry Matter; (**c**) Vitamin C; (**d**) Total Acid; (**e**) Soluble Sugar; (**f**) Soluble Solids; (**g**) Protein; (**h**) β-Carotene; (**i**) Total 16 Amino Acids.In the boxplots, the horizontal line within the box represents the median, the upper and lower edges of the box represent the upper and lower quartiles, and the whiskers denote the upper and lower edges of the data. The corresponding mean values are presented in Table 2.

To investigate the impact of different geographical origins on the quality of cherry tomatoes [25], this study selected cherry tomato samples from Beijing and Shandong and performed independent samples *t*-tests and boxplot distribution analyses on nine core quality indices, including Lycopene, Dry Matter, Vc, Total Acid, and Soluble Sugar (Table 2, Figure 2).

#### 3.2.1. Analysis of Basic Flavor Substances and Texture Indices

Statistical results indicated that cherry tomatoes from the Shandong region were significantly superior to those from the Beijing region (*p* < 0.05) in key indices determining flavor intensity and fruit fullness.

Specifically, the Dry Matter content of the Shandong group was 7.82 ± 1.45, extremely significantly higher than the 6.29 ± 1.68% of the Beijing group (*p* < 0.001). The Soluble Solids (TSS) content in the Shandong group (6.69%) was also significantly higher than in the Beijing group (5.34%) (*p* < 0.001).

Regarding flavor presentation, the difference in Total Acid was most significant (*p* < 10^−13^), with the Shandong group mean (5.16 g/kg) far exceeding the Beijing group (3.30 g/kg). Boxplots showed almost no overlap between the two distributions, indicating a stronger sweet-sour flavor profile in Shandong samples.

Notably, although Soluble Sugar was significantly higher in the Shandong group (6.66%) than in the Beijing group (3.01%, *p* = 0.026), the Shandong samples showed much greater dispersion, with a standard deviation of 12.32 and several extreme outliers in the boxplot. In contrast, the Beijing group exhibited a more concentrated and consistently lower sugar distribution, indicating greater uniformity but fewer high-sugar samples.

Additionally, the Protein content (0.93%) and total content of 16 amino acids (0.73 g/100 g) in the Shandong group were also significantly higher than in the Beijing group (*p* < 0.01), further confirming the advantage of Shandong cherry tomatoes in accumulating umami substances.

#### 3.2.2. Analysis of Functional Antioxidant Components

Regarding pigment-related and antioxidant components, the samples from the two regions showed asynchronous trends. Lycopene, as the primary colorant and functional component of tomatoes, had a mean value of 62.46 mg/kg in the Shandong group, significantly higher than the 49.99 mg/kg in the Beijing group (*p* = 0.018). Combined with the boxplots, the Shandong group showed a higher maximum value and wider interquartile range (Figure 2b), indicating that this region is more conducive to pigment synthesis and accumulation, likely resulting in fruits with a redder appearance.

However, no significant differences were observed between the two regions for Vc and β-Carotene contents. The Vc contents for the Beijing and Shandong groups were 23.21 mg/100 g and 24.23 mg/100 g, respectively, with a *p*-value of 0.63 (*p* > 0.05); the *p*-value for β-Carotene was 0.06 (*p* > 0.05). These results suggest that Vc synthesis is more stable than sugars and acids under the production conditions of both regions. Geographical differences did not significantly affect Vc in cherry tomatoes.

### 3.3. Comprehensive Effect Analysis of Regional Environment on Quality Characteristics of Different Tomato Types

As noted in Section 2.1, regional groups differ in both environmental conditions and varietal composition; accordingly, the ‘regional effects’ discussed below should be understood as composite effects that cannot be attributed to geography alone. Combining the test data of regular tomatoes and cherry tomatoes, this study not only verified varietal differences but also revealed consistent and statistically significant regional differences in multiple quality indices across both tomato types. Comparative analysis across fruit types demonstrated distinct quality formation mechanisms and regional profiles for Beijing and Shandong.

#### 3.3.1. Consistency of Regional Flavor Orientation and Dry Matter Accumulation

In both regular and cherry tomatoes, samples from the Shandong region exhibited a highly consistent characteristic of high Dry Matter and high flavor, establishing its status as a quality-oriented production region. Data showed that Shandong samples were significantly higher than their Beijing counterparts (*p* < 0.05) in three core indices: Dry Matter, Soluble Solids, and Total Acid content. This cross-variety advantage suggests that the light and thermal resources or cultivation management modes in Shandong are extremely favorable for the transport and concentration of photosynthates into the fruit [26]. The resulting fruit characteristics are manifested as dense flesh, intense flavor, and efficient accumulation of pigment-related and flavor-related components.

#### 3.3.2. Population Differentiation Characteristics of Sugar Accumulation

Although the Shandong region was generally superior to Beijing in sugar indices, the two fruit types revealed distinct pathways through which this quality advantage was expressed. In regular tomatoes, the sugar advantage of the Shandong group manifested as a population-wide upward shift in the entire distribution (Figure 1e), suggesting a collective increase in the accumulation of sugar-related components, possibly attributed to the more favorable light and thermal conditions of open-field or semi-facility production environments [27]. In cherry tomatoes, by contrast, the sugar advantage was largely concentrated in a subset of ultra-high sugar samples (Figure 2e), while the core distribution of the two groups showed comparatively smaller differences. This divergence suggests different improvement pathways across fruit types. Regular tomatoes likely benefit from environment-driven, population-level enrichment of primary quality-related components [28,29,30]. In cherry tomatoes, high-end quality is mainly shaped by varietal differentiation, particularly specialized high-sugar germplasms adapted to local conditions [31]. Taken together, these contrasting patterns suggest that breeding and cultivation strategies for quality improvement should be tailored to fruit type: for regular tomatoes, optimizing the regional production environment may yield broad population-level gains [32], while for cherry tomatoes, targeted selection and promotion of elite high-sugar varieties will be of greater importance [33].

#### 3.3.3. Water Characteristics and Nutrient Retention Under Nutritional Supply-Oriented Mode

Samples from the Beijing region exhibited typical yield and nutritional supply-oriented characteristics, marked significantly by extremely high water content in both tomato types. This is typically associated with high-yield facility cultivation and high water/fertilizer management strategies in urban agriculture [34]. Although this dilution effect led to a relative reduction in the concentration of flavor substances [35], the Beijing region demonstrated certain resilience in Vc retention: in regular tomatoes, the Vc content in the Beijing group was even slightly superior to the Shandong group (Figure 1h); in cherry tomatoes, no significant difference was found (Figure 2h). This indicates that in a water-replete growth environment, while the accumulation of flavor substances is affected, the accumulation and maintenance of basic vitamins such as Vc remain relatively stable and are not negatively inhibited.

### 3.4. Regional Quality Clustering Characteristics Based on Principal Component Analysis (PCA)

**Figure 3:**
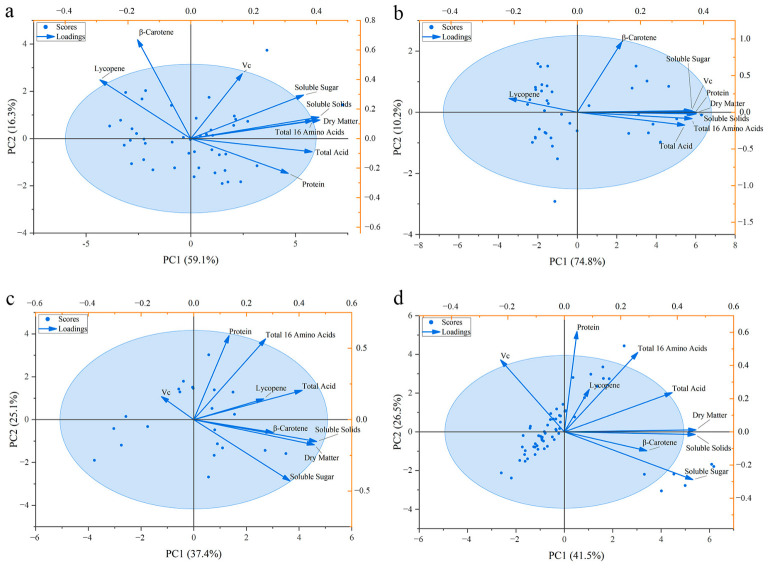
PCA score plot of tomato varieties from different geographical origins: (**a**) Beijing Regular Tomato, (**b**) Beijing Cherry Tomato, (**c**) Shandong Regular Tomato, (**d**) Shandong Cherry Tomato.

To overcome the limitations of single-index analysis, this study employed Principal Component Analysis (PCA) to conduct dimensionality reduction and clustering analysis on the quality data of regular and cherry tomatoes. Figure 3 display Biplots of PC1 (First Principal Component) and PC2 (Second Principal Component), revealing clear regional clustering patterns and variable contribution relationships.

#### 3.4.1. Multivariate Association of Quality Indices

The results indicated that the cumulative variance explained by the principal components of Beijing regular (Figure 3a) and cherry tomatoes (Figure 3b) surpassed 50%, effectively explaining most of the data variation. In all PCA Score Plots, sample points were not randomly distributed but exhibited obvious regional clustering characteristics. Sample points representing the Shandong region were primarily concentrated in the positive direction of PC1, while Beijing samples were distributed in the negative direction or near the central zero point. This clear spatial separation indicates that the regional environment has a decisive shaping effect on the overall quality-related compositional profile of tomatoes, and products from the two regions belong to two distinct populations in the multidimensional quality space [36]. For Shandong regular and cherry tomatoes (Figure 3c,d), the cumulative variance explained by PC1 and PC2 was below 60%, indicating moderate explanatory power. This is likely attributable to greater compositional heterogeneity within the Shandong population driven by wider varietal diversity—particularly the presence of extreme high-sugar outliers in cherry tomatoes. Despite the moderate explained variance, the directional separation of sample clusters along PC1 remains interpretable, as it consistently reflects the positive co-loading of the four flavor-texture indices.

#### 3.4.2. Definition and Drivers of the Flavor-Texture Axis (PC1)

Observing the direction and length of vectors in the Loading Plots, it can be found that PC1 is primarily driven jointly by Dry Matter, Soluble Solids, Total Acid, and Soluble Sugar. The vectors of these indices clustered tightly and pointed in the same direction, indicating a strong positive correlation.

This pattern suggests that PC1 may be operationally interpreted as a composite axis reflecting the co-variation in flavor- and texture-related indices, although PCA is an exploratory tool and the biological significance of principal components should not be over-interpreted. It should be noted that the descriptive labels assigned to principal components (e.g., ‘flavor–texture axis’) are operational summaries of the observed loading patterns rather than validated biological constructs. The high scores of Shandong samples on the PC1 axis reaffirm their overall advantage of high Dry Matter and high flavor accumulation. Quality indices do not vary independently in the multivariate space; higher Dry Matter is often accompanied by higher levels of multiple flavor-related indices within the sampled populations. The numerical loading values for all variables on PC1 and PC2 for each analysis group are provided in Table S2.

#### 3.4.3. Divergent Loading Pattern of Vitamin C Relative to Flavor-Texture Indices

Notably, in several Biplots, the loading vector of Vc exhibited a behavioral pattern distinct from other core quality indices.

In some plots (Figure 3c,d), the direction of the Vc vector was nearly perpendicular or even opposite to the sugar-acid complex vector. This loading pattern is consistent with the hypothesis that Vc accumulation may be physiologically relatively independent and may not follow the pattern in which Dry Matter concentration brings an across-the-board nutritional increase, although confirmation would require targeted physiological experiments. This multidimensional analysis result corroborates the univariate analysis conclusions in Section 3.2 and Section 3.3: although the Shandong region dominates in flavor and texture indices, it does not show the same coordinated increase in Vc retention, a specific nutrient, and is even at parity or a disadvantage compared to the high-water samples from Beijing.

#### 3.4.4. Differences in Clustering Morphology Between Regular and Small Fruit Types

Comparing the PCA plots of regular tomatoes and cherry tomatoes, a difference in clustering tightness was observed. The clustering of regular tomato sample points was relatively compact, indicating good quality consistency and stable regional characteristics; whereas cherry tomato sample points showed greater dispersion along the PC2 axis. Combined with loading vector analysis, this can be largely attributed to the extremely high sugar and Lycopene contents in some cherry tomato samples, which stretched the data in the multidimensional space. This observation is consistent with more intense varietal differentiation within the cherry tomato group, where specific high-sugar samples may act as outlier forces contributing to the observed quality variation.

### 3.5. Correlation Analysis of Quality Indices and Compositional Relationships

**Figure 4:**
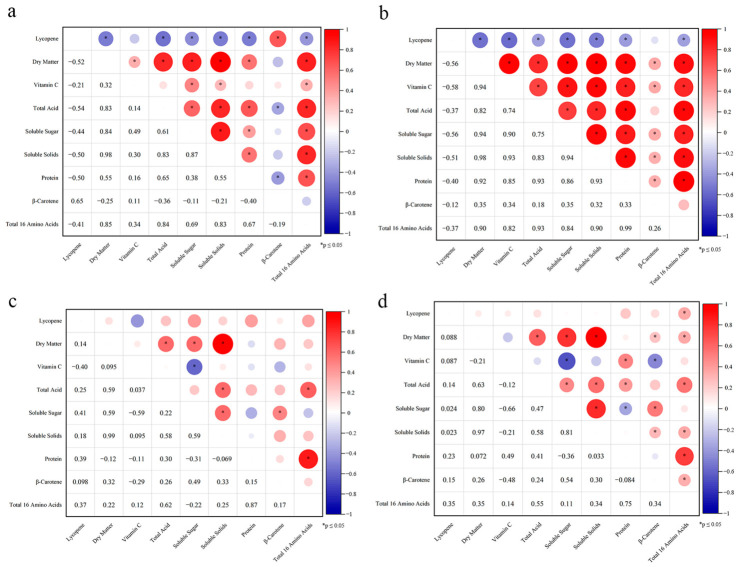
Correlation heatmap of major nutritional components in different tomato cultivars across various geographical regions: (**a**) Beijing Regular Tomato, (**b**) Beijing Cherry Tomato, (**c**) Shandong Regular Tomato, (**d**) Shandong Cherry Tomato.

To explore the associations among tomato quality indices, this study conducted Pearson correlation analysis on samples from different origins and varieties. The heatmaps in Figure 4 visually display the magnitude and direction of correlation coefficients (*r*) between indices, revealing the coupling modes of flavor-nutrition-texture under different environments.

#### 3.5.1. Coordinated Accumulation Pattern Associated with Dry Matter Concentration Effect

In sample groups exhibiting high-quality characteristics, a strong pattern of positive associations was observed (Figure 4c). The heatmap showed that Dry Matter, as a core driving factor, had highly significant positive correlations (*r* > 0.8, *p* < 0.01) with Vc, Total Acid, Soluble Sugar, Soluble Solids, Protein, and total amino acids.

This strong positive network is consistent with a Dry Matter concentration effect, in which reduced fruit water leads to elevated cell sap concentration and synchronous enrichment of flavor and nutritional components [37].

#### 3.5.2. Divergent Association Patterns Between Sugar/Acid Accumulation and Vitamin C

In contrast to the coordinated accumulation pattern, Vc of Beijing Cherry Tomato showed a relatively independent accumulation pattern and even appeared significantly negatively correlated (Figure 4b).

Data showed a significant negative correlation between Vc and Soluble Sugar (*r* = −0.59, *p* < 0.05) as well as Soluble Solids in Beijing cherry tomatoes (Figure 4b). This association remained significant after Benjamini–Hochberg False Discovery Rate correction (Table S3), suggesting a possible compositional trade-off or asynchronous dilution effect in water-replete growth environments [38]. However, this finding is correlational in nature; direct mechanistic confirmation would require metabolic flux analysis or enzymatic assays.

#### 3.5.3. Coupling of Flavor Substances: Strong Association Between Sugar and Acid

In all correlation heatmaps, Soluble Sugar and Soluble Solids consistently maintained an extremely high positive correlation, verifying the reliability of Soluble Solids as a convenient indicator for predicting tomato sweetness. Meanwhile, Sugar and Total Acid were also positively correlated in most cases, especially closely associated in the high-quality group. This indicates that the flavor formation of premium tomatoes is not a single-dimensional increase in sugar but a dynamic balance of the sugar-acid ratio at a higher level. High sugar is often accompanied by high acid, thereby constructing a rich and mellow typical tomato flavor, avoiding the cloying sweetness of pure high sugar or the harshness of pure high acid.

#### 3.5.4. Relative Independence of Pigment-Related Components

Notably, the correlations of Lycopene and β-Carotene with other major physicochemical indices showed considerable fluctuation. In Figure 4d, the correlation between pigment content and Dry Matter or sugar degree was weak (*r* < 0.4) or insignificant. This suggests that the pigment biosynthetic processes determining fruit color are relatively independent of the sugar/acid-related processes determining mouthfeel [39]. Whether a fruit is red is predominantly regulated by genotype and light/temperature, and there is no absolute binding relationship with whether the fruit is fleshy and juicy or sweet and sour.

### 3.6. Analysis of Amino Acid Component Characteristics and Taste Profile Differences

#### 3.6.1. Clustering Characteristics and Distribution of Amino Acid Components

**Figure 5:**
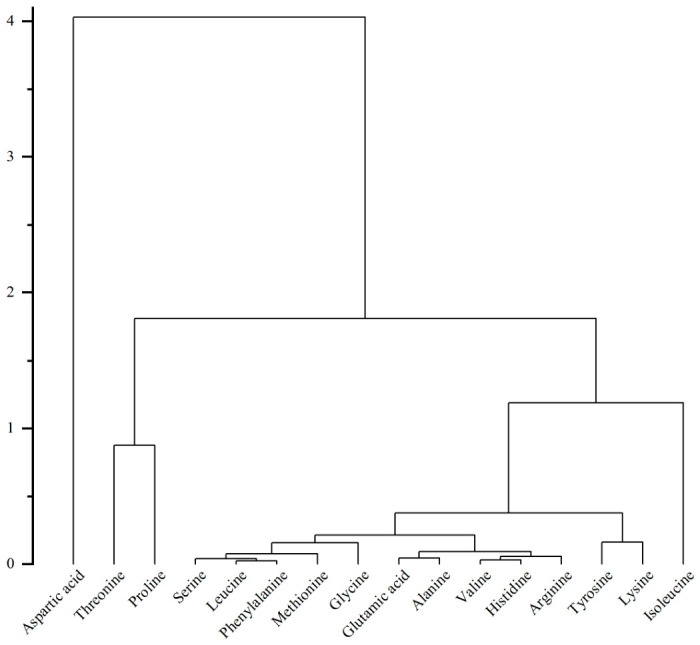
Hierarchical clustering dendrogram of amino acid composition in 165 tomato samples from Beijing and Shandong.

Hierarchical clustering analysis based on Euclidean distance classified the 16 amino acids into three distinct accumulation clusters (Figure 5). The first cluster, representing high-abundance components, consisted of aspartic acid, threonine, and proline. Notably, aspartic acid exhibited the greatest Euclidean distance (>4.0) from other components, demonstrating the most significant accumulation dominance. The second cluster comprised the majority of medium-abundance amino acids, including glutamic acid, serine, and leucine, while the third cluster consisted of low-abundance amino acids such as isoleucine and lysine.

**Figure 6:**
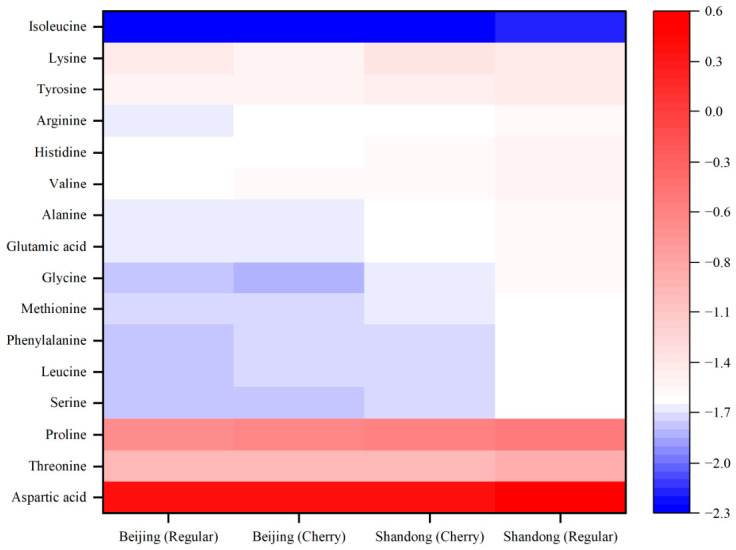
Clustered heatmap of 16 amino acids in tomato cultivars from different geographical origins.

The heatmap further visualized the distribution differences in amino acid content across varying samples (Figure 6). Horizontally, the accumulation patterns remained consistent across all sample groups, with aspartic acid and glutamic acid consistently maintaining high content levels, whereas isoleucine consistently remained at the lowest level. Vertically, however, significant variations in overall abundance were observed among origins. The Shandong Regular tomato samples were predominantly characterized by red high-value regions in the heatmap, indicating a globally higher absolute content of various amino acids. In contrast, samples from the Beijing region (both Regular and Cherry) were mainly concentrated in lighter or blue regions, reflecting a relatively lower overall accumulation of amino acids.

#### 3.6.2. Glutamate-Dominated Umami Fingerprint Characteristics

A highly conserved pattern was observed across all heatmaps: in all samples, Glutamate (Glu) and Aspartate (Asp) were consistently located in the high-abundance range, while other essential amino acids were mostly in the low-abundance range.

Glutamate is a well-characterized umami amino acid and is widely regarded as a primary contributor to tomato flavor. The heatmap results suggest that regardless of origin, tomatoes appear to retain an amino acid profile dominated by glutamate. This specific amino acid composition may contribute to unique meatiness and savoriness in sensory perception. However, formal sensory validation remains necessary to confirm these hypothesized flavor-enhancing effects. Furthermore, although the component proportions are similar, the difference in absolute content becomes key to distinguishing regional quality.

#### 3.6.3. Interaction of Sweet-Umami Flavors

From the perspective of taste chemistry, amino acids and the sugar-acid system may interact synergistically. The high glutamate (umami) in the Shandong group coexists with the high Soluble Sugar (sweetness) and high organic acid (sourness) analyzed earlier. Literature suggests that the umami threshold of monosodium glutamate may be lowered and sensory intensity potentially enhanced in the presence of salt or acid.

Based on the chemical composition data, the coexistence of high glutamate with elevated Soluble Sugar and organic acid concentrations in Shandong samples is chemically consistent with a potential flavor enhancement effect, as documented in the literature [40]. However, as no direct sensory panel evaluation was conducted in this study, the perceptual consequences of these chemical differences remain to be confirmed by future sensory validation studies.

## 4. Discussion

### 4.1. Source–Sink Balance and Carbon Allocation

Because varietal composition was not standardized between the Beijing and Shandong groups, the regional differences discussed below should be interpreted as composite outcomes of environmental conditions, cultivation practices, and varietal composition, rather than effects of geographical origin alone. Accordingly, the interpretations proposed here are presented as working hypotheses derived from field-survey patterns rather than direct causal demonstrations.

The physicochemical indices measured in this study cover key nodes of primary and secondary metabolism. Taken together, the results suggest that the observed differences between the two regional groups are consistent with patterns related to source–sink relations and carbon allocation during fruit development.

Tomato fruit quality is fundamentally influenced by the transport and conversion capacity of photosynthates (source) to the fruit (sink). The synchronous enrichment of high Soluble Sugar (carbon source) and high Lycopene (secondary metabolite) exhibited by Shandong samples is consistent with observations of enhanced carbon allocation or assimilate accumulation [41], as reported in tomato systems under reduced water supply [42], although direct flux analysis was not performed in this study. From a biochemical pathway perspective, sugar is not only the source of sweetness but also provides the carbon skeleton and energy basis for pigment synthesis. It has been proposed that only when primary carbon assimilation is sufficient may sufficient acetyl-CoA enter the isoprenoid pathway, thereby supporting Lycopene synthesis [43]. Beijing samples, due to excessive water content, have relatively lower intracellular sugar concentrations, potentially limiting the allocation of carbon skeletons to pigment biosynthetic pathways, resulting in insufficient accumulation of fruit color and flavor substances.

By contrast, the lower Dry Matter, Soluble Solids, and sugar levels observed in the Beijing groups are compatible with a more water-replete production pattern. Under such conditions, fruit composition may be diluted, leading to lower concentrations of flavor-related metabolites even when basic nutritional components are maintained.

### 4.2. Potential Association of TCA-Cycle-Related Processes with Flavor Formation

Correlation analysis and amino acid heatmaps were consistent with a strong positive correlation between organic acids and amino acids. This is often explained by the tricarboxylic acid (TCA) cycle’s role as not only the core of energy metabolism but also the hub connecting carbon metabolism and nitrogen metabolism [44,45]. Intermediates of the TCA cycle are direct precursors for the synthesis of glutamate and aspartate. The high Total Acid content in Shandong samples suggests that TCA-related processes may be more active in Shandong samples, consistent with the known role of TCA intermediates as precursors for glutamate and aspartate biosynthesis for nitrogen assimilation, thereby potentially promoting the substantial synthesis of umami amino acids. This coordinated compositional pattern of high sugar → high acid → high amino acids explains why high-quality tomatoes often possess a composite mouthfeel of intense sweetness, crisp sourness, and savoriness.

In regular tomatoes, the Shandong group showed simultaneously higher Total Acid and Total 16 Amino Acids than the Beijing group. A similar co-occurrence was also observed in cherry tomatoes, where the Shandong group exhibited higher acidity, Protein, and total amino acids. These descriptive findings are consistent with the possibility that stronger accumulation of organic acids may support, or at least accompany, enhanced synthesis of amino acids such as glutamate and aspartate, which are major contributors to tomato taste chemistry.

Rather than demonstrating a direct metabolic cascade, the present data indicate a coordinated compositional pattern in which sugar-, acid-, and amino acid-related traits tend to increase together in the higher-quality sample groups.

### 4.3. Relative Independence and Environmental Responsiveness of Vitamin C Accumulation

Notably, Vc showed a relatively independent accumulation pattern among the measured quality traits. Although Vc is also synthesized from hexoses (glucose/galactose) via the L-galactose pathway, its accumulation is specifically regulated by light intensity and oxidative stress [46] and is not completely synchronous with the main sugar-acid pathway [47]. The fact that Beijing samples maintained Vc levels comparable to or even slightly higher than Shandong samples despite having sufficient water and lower Dry Matter suggests that Vc synthesis may be less affected by the dilution effect, or it may represent a basal homeostasis maintained by plants to cope with specific environmental pressures in facility cultivation environments. This also suggests that when improving quality through agronomic means, the pathways for enhancing sugar-acid flavor and Vc nutrition may need to be regulated separately.

This interpretation is also consistent with the exploratory PCA loading patterns, in which the Vc vector was separated from the sugar–acid-related loadings in some biplots. However, because PCA is an exploratory method, this pattern should be interpreted only as evidence of different covariance structures, not as proof of an independent biological pathway by itself.

### 4.4. Dry Matter Concentration Effect as the Physiological Basis for Quality Differentiation

These data support the Dry Matter concentration effect as a primary physiological contributor to quality differentiation, consistent with previous studies [48,49]. The distinct light and thermal advantages in the Shandong region likely promoted the efficient transport and accumulation of photosynthates to the fruit sink, contributing to a compositional framework characterized by high sugar and high acid. This enhanced carbon allocation pattern not only improved the sensory sugar-acid ratio of the fruit but also provided sufficient substrates for secondary metabolism, thereby potentially driving the synchronous enrichment of functional pigments such as Lycopene and contributing to an overall coordinated improvement in the fruit’s physicochemical quality.

Higher Dry Matter is generally associated with lower relative water content and a firmer, denser fruit texture. In cherry tomatoes, the higher Dry Matter and Soluble Solids observed in the Shandong group are similarly consistent with a more concentrated compositional profile and potentially stronger sensory intensity. These patterns do not establish a single physiological mechanism, but they support the view that fruit water relations and assimilate concentration are important components of the regional differences observed here.

### 4.5. Coordinated Carbon–Nitrogen Compositional Patterns in Flavor Formation

Correlation heatmaps confirmed that in high-quality tomatoes, the associations among organic acids and amino acids are consistent with the TCA cycle functioning as a hub connecting carbon and nitrogen metabolism, potentially supporting the accumulation of umami amino acids such as glutamate and aspartate. The coupled high sugar/acid and high amino acid pattern in Shandong samples may underlie their rich taste and complex flavor layers. It also suggests a generally higher coordination of quality-related components, consistent with multi-parameter quality studies [50,51].

The higher Lycopene and β-Carotene contents in Shandong regular tomatoes are consistent with fruits having deeper red coloration and greater carotenoid accumulation. In cherry tomatoes, Lycopene was also higher in the Shandong group, whereas β-Carotene did not differ significantly between regions. These pigment differences may reflect the combined influence of varietal background and production environment, including light and temperature conditions known to affect carotenoid biosynthesis [27,28]. However, because genotype and region were confounded in this survey, the relative contribution of each factor cannot be separated here.

### 4.6. Fruit Type-Specific Quality Formation Pathways

Analysis of different fruit types further revealed differences in quality formation pathways [52,53]. The quality differences in common tomatoes manifested predominantly as a collective shift driven by the regional environment; whereas cherry tomatoes showed stronger germplasm specificity, with the advantages of the Shandong region being primarily driven by certain outlier germplasms with extremely high sugar and Lycopene content. This suggests that the high-end development of cherry tomatoes depends not only on the environmental background but also on the expression potential of specific elite varieties under suitable conditions.

In regular tomatoes, the higher sugar-related values in the Shandong group appeared to reflect a broader upward shift in the distribution. This pattern is consistent with a group-level difference in compositional concentration. In cherry tomatoes, by contrast, the Shandong group showed much larger variability in Soluble Sugar, including multiple high-value outliers. This suggests greater within-group heterogeneity and may indicate the presence of specialized high-sugar germplasm in part of the sample set.

Importantly, because varietal composition differed between regions, these fruit-type-specific patterns should not be attributed solely to environmental conditions. Instead, they likely reflect different combinations of production environment and cultivar structure in the two regional sample populations.

The broader dispersion of cherry tomato traits, including sugar and Lycopene, also agrees with the more scattered clustering seen in the exploratory PCA plots. Again, this should be interpreted as a pattern of multivariate heterogeneity rather than evidence for a discrete biological class.

### 4.7. Limitations

Several limitations of this study should be acknowledged. First, the field survey design precluded full varietal standardization; the observed regional differences represent the integrated effect of environment, cultivation practice, and genotype and cannot be attributed solely to geographical origin. Second, no direct sensory evaluation was performed; all flavor quality inferences are based on chemical composition data and should be validated by sensory panel studies. Third, the interpretations regarding underlying mechanisms proposed in this study (e.g., enhanced carbon flux, TCA cycle activity) are inferences from correlation analyses and are not directly supported by metabolic flux measurements, enzymatic assays, or gene expression data. Fourth, variety information for a proportion of samples was unavailable due to the commercial production survey design. Future studies employing controlled genotype × environment experiments with defined agronomic conditions and sensory validation are needed to substantiate and refine the hypotheses raised here.

## 5. Conclusions

Based on a multidimensional compositional analysis strategy, this study supports the hypothesis that the integrated effects of regional production environment, predominant cultivation system, and varietal composition contribute to compositional differentiation in tomato quality between Beijing and Shandong. However, because varietal composition was not standardized between regions, the observed differences cannot be attributed solely to environmental factors; causal disentanglement will require controlled genotype × environment experiments. Principal component analysis showed clear regional clustering of tomato samples from Beijing and Shandong, reflecting compositional differences in quality-related traits between the two production systems. Samples from the Shandong region established a core status as a high-flavor-density area due to significantly superior Dry Matter, Soluble Solids, and Total Acid content, whereas the Beijing region presented typical characteristics of high-water nutritional supply-oriented facility agriculture.

Unlike sugars, acids, and flavor substances, which are subject to the water dilution effect, Vc showed a relatively independent pattern. Beijing fruits maintained comparable or even slightly higher Vc levels despite lower Dry Matter. This suggests that water management in facility systems may dilute flavor concentration without necessarily reducing Vc accumulation. Overall, nutrient-supply-oriented cultivation can maintain basic functional nutrition.

In summary, this study clarifies that the abundance and coordination among quality-related components are key factors contributing to quality differentiation by origin. The research defines the differentiated compositional characteristics of flavor-oriented (Shandong) and nutritional supply-oriented (Beijing) production, providing compositional evidence that may support future region-specific breeding and cultivation strategies: These findings should be regarded as working hypotheses warranting further investigation under controlled conditions. Given that varietal composition was confounded with region in the present survey design, high dry-matter varieties may be particularly suited to the light-resource-rich production environments of Shandong, while urban facility systems in Beijing may benefit from agronomic strategies that preserve ascorbic acid without compromising yield. Verification through controlled genotype × environment experiments using standardized cultivar panels is essential before translating these findings into specific breeding or cultivation recommendations.

## Figures and Tables

**Table 1:** Comparison of quality and nutritional indicators between Beijing and Shandong regular tomato groups.

Traits	Beijing	Shandong	*p*-Value
Lycopene (mg/kg)	38.22 ± 23.9 **	67.32 ± 32.1	0.0012
Dry Matter (%)	6.73 ± 1.3 ***	8.88 ± 1.8	3.0 × 10^−5^
Vitamin C (mg/100 g)	17.79 ± 5.7 *	13.98 ± 5.1	0.0483
Total Acid (g/kg)	4.08 ± 1.1 ***	5.57 ± 1.1	6.0 × 10^−5^
Soluble Sugar (%)	3.41 ± 0.8 **	8.66 ± 7.0	0.0025
Soluble Solids (%)	5.80 ± 1.4 ***	8.04 ± 1.8	6.0 × 10^−6^
Protein (%)	0.79 ± 0.1	0.82 ± 0.2	0.6132
β-Carotene (mg/kg)	2.32 ± 0.5 ***	3.76 ± 1.6	0.001
Total 16 Amino Acids (g/100 g)	0.63 ± 0.1 ***	0.82 ± 0.2	0.0001

Values are means  ±  SD. Significance: not significant (*p* > 0.05); * *p* < 0.05; ** *p* < 0.01; *** *p* < 0.001. Statistical differences between the Beijing and Shandong regular tomato groups were evaluated using independent samples *t*-tests.

**Table 2:** Comparison of quality and nutritional indicators between Beijing and Shandong cherry tomato groups.

Parameters	Beijing	Shandong	*p*-Value
Lycopene (mg/kg)	49.99 ± 23.95 *	62.46 ± 27.02	0.0182
Dry Matter (%)	6.29 ± 1.68 ***	7.82 ± 1.45	1.27 × 10^−5^
Vitamin C (mg/100 g)	23.21 ± 10.73	24.23 ± 9.67	0.63
Total Acid (g/kg)	3.30 ± 0.98 ***	5.16 ± 1.01	4.13 × 10^−14^
Soluble Sugar (%)	3.01 ± 1.19 *	6.66 ± 12.32	0.0259
Soluble Solids (%)	5.34 ± 1.49 ***	6.69 ± 1.42	2.48 × 10^−5^
Protein (%)	0.76 ± 0.25 ***	0.93 ± 0.17	3.04 × 10^−4^
β-Carotene (mg/kg)	2.56 ± 0.46	3.00 ± 1.73	0.0625
Total 16 Amino Acids (g/100 g)	0.61 ± 0.20 **	0.73 ± 0.18	0.0051

Values are means  ±  SD. Significance:not significant (*p* > 0.05); * *p* < 0.05; ** *p* < 0.01; *** *p* < 0.001. Statistical differences between the Beijing and Shandong cherry tomato groups were evaluated using independent samples *t*-tests.

## Data Availability

The original contributions presented in this study are included in the article/Supplementary Material. Further inquiries can be directed to the corresponding authors.

## Supplementary Materials

The following supporting information can be downloaded at: https://www.mdpi.com/article/10.3390/foods15101816/s1, Table S1: Sample information and quality parameters of 165 tomato accessions from Beijing and Shandong; Table S2: Principal Component Analysis (PCA) loading matrices for Beijing regular tomato; Table S3: Principal Component Analysis (PCA) loading matrices for Beijing cherry tomato; Table S4: Principal Component Analysis (PCA) loading matrices for Shandong regular tomato; Table S5: Principal Component Analysis (PCA) loading matrices for Shandong cherry tomatoes; Table S6: Pearson correlation coefficients and FDR-adjusted *p*-values for quality parameters: Beijing regular tomato; Table S7: Pearson correlation coefficients and FDR-adjusted *p*-values for quality parameters: Beijing cherry tomato; Table S8: Pearson correlation coefficients and FDR-adjusted *p*-values for quality parameters: Shandong regular tomato; Table S9: Pearson correlation coefficients and FDR-adjusted *p*-values for quality parameters: Shandong cherry tomato.

## Abbreviations

The following abbreviations are used in this manuscript:

FAO	Food and Agriculture Organization
G × E	Genotype × Environment
PSY1	Phytoene Synthase 1
HPLC	High-Performance Liquid Chromatography
TSS	Total Soluble Solids
GB	Guobiao/National Standards of China
SD	Standard Deviation
PCA	Principal Component Analysis
HCA	Hierarchical Cluster Analysis
PC1	First Principal Component
PC2	Second Principal Component
Vc	Vitamin C
TCA	Tricarboxylic Acid Cycle
Glu	Glutamate
Asp	Aspartate
