# Supplementary material for: Nutritional Supply vs. Flavor Quality: Characterizing the Physicochemical Properties and Amino Acid Profiles of Tomatoes from Beijing and Shandong
Source: Foods. 2026 May 20;15(10):1816. doi: 10.3390/foods15101816 (PMC13205225; doi:10.3390/foods15101816)
Supplement: Supplementary file 1 [file foods-15-01816-s001.zip › foods-4247125-supplementary.pdf]

**Supplementary Material**

**Table S1.** Sample Information and Quality Parameters of 165 Tomato Accessions from Beijing and Shandong.

| Sample ID | Region | City/County | Fruit Type | Cultivation System | Collection Date | Variety Name |
|---|---|---|---|---|---|---|
| R001 | Beijing | Changping | Regular | Protected facility | 2022.06.17 | 24609 |
| R002 | Beijing | Changping | Regular | Protected facility | 2023.06.27 | Caifan T99AW |
| R003 | Beijing | Changping | Regular | Protected facility | 2022.06.24 | Caifan XX445 |
| R004 | Beijing | Changping | Regular | Protected facility | 2022.06.17 | Chunshangye 2 |
| R005 | Beijing | Changping | Regular | Protected facility | 2022.07.04 | Fenrun 1910 |
| R006 | Beijing | Changping | Regular | Protected facility | 2022.06.17 | Fengying 7391 |
| R007 | Beijing | Changping | Regular | Protected facility | 2023.07.01 | Gaoshan Baoshi |
| R008 | Beijing | Changping | Regular | Protected facility | 2022.07.04 | Guanshu |
| R009 | Beijing | Changping | Regular | Protected facility | 2022.07.04 | Jiafan 3 |
| R010 | Beijing | Changping | Regular | Protected facility | 2023.06.27 | Jingcai 8 (No.5) |
| R011 | Beijing | Changping | Regular | Protected facility | 2022.07.04 | Jingcai 8 No.1 |
| R012 | Beijing | Changping | Regular | Protected facility | 2023.06.27 | Jingcai 8 No.2 |
| R013 | Beijing | Changping | Regular | Protected facility | 2023.07.22 | Jingfan 901 (No.60) |
| R014 | Beijing | Changping | Regular | Protected facility | 2022.06.17 | Lengyanhong 6722 |
| R015 | Beijing | Changping | Regular | Protected facility | 2023.07.22 | Lusheng Fulu |
| R016 | Beijing | Changping | Regular | Protected facility | 2023.07.22 | Lusheng Fuwa |
| R017 | Beijing | Changping | Regular | Protected facility | 2023.06.27 | Pinfan CY128 |
| R018 | Beijing | Changping | Regular | Protected facility | 2023.07.22 | Qingfan 2 |
| R019 | Beijing | Changping | Regular | Protected facility | 2022.06.24 | Qinglian 190 |

| | | | | | | |
|---|---|---|---|---|---|---|
| R020 | Beijing | Changping | Regular | Protected facility | 2022.06.24 | Ruifei 2 |
| R021 | Beijing | Changping | Regular | Protected facility | 2023.07.22 | Shenfen 301 |
| R022 | Beijing | Changping | Regular | Protected facility | 2022.07.04 | Shixiang Yilin |
| R023 | Beijing | Changping | Regular | Protected facility | 2023.07.22 | Sijiali |
| R024 | Beijing | Changping | Regular | Protected facility | 2022.06.17 | Suan Tian Baozhu |
| R025 | Beijing | Changping | Regular | Protected facility | 2023.06.27 | Tianwei 215 |
| R026 | Beijing | Changping | Regular | Protected facility | 2022.06.24 | Tian Cuicui (No.25) |
| R027 | Beijing | Changping | Regular | Protected facility | 2023.06.27 | Weiduomei 9 (No.2) |
| R028 | Beijing | Changping | Regular | Protected facility | 2022.06.24 | Weiqin 1 |
| R029 | Beijing | Changping | Regular | Protected facility | 2023.07.01 | Xinpingguo ×8 (No.2) |
| R030 | Beijing | Changping | Regular | Protected facility | 2022.07.04 | Xiuming |
| R031 | Beijing | Changping | Regular | Protected facility | 2023.07.01 | Yadianna |
| R032 | Beijing | Changping | Regular | Protected facility | 2022.06.17 | Yuejia 1 |
| R033 | Beijing | Changping | Regular | Protected facility | 2023.07.22 | Zhongying 1001 |
| R034 | Beijing | Changping | Regular | Protected facility | 2023.06.27 | B8181 |
| R035 | Beijing | Changping | Regular | Protected facility | 2022.06.17 | Unknown (B8182) |
| R036 | Beijing | Changping | Regular | Protected facility | 2022.06.24 | B8183 |
| R037 | Beijing | Changping | Regular | Protected facility | 2022.06.17 | B8184 |
| R038 | Beijing | Changping | Regular | Protected facility | 2023.07.01 | B8185 |
| R039 | Beijing | Changping | Regular | Protected facility | 2022.06.24 | Fengshou 128 |
| R040 | Beijing | Changping | Regular | Protected facility | 2022.06.17 | Liaoning 11 |
| R041 | Beijing | Changping | Regular | Protected facility | 2023.07.01 | Provence |

| | | | | | | |
|---|---|---|---|---|---|---|
| R042 | Beijing | Changping | Regular | Protected facility | 2023.07.01 | Xilaide 1 |
| R043 | Beijing | Changping | Regular | Protected facility | 2022.06.24 | Zhuofen 1 |
| R044 | Beijing | Changping | Regular | Protected facility | 2022.06.17 | Zhuofen 2 |
| R045 | Shandong | Shouguang | Regular | Open field | 2022.06.17 | Gaotang Huangfei |
| R046 | Shandong | Shouguang | Regular | Open field | 2022.06.17 | Caomeiguo |
| R047 | Shandong | Shouguang | Regular | Open field | 2023.07.01 | Jingcai |
| R048 | Shandong | Shouguang | Regular | Open field | 2022.06.17 | Caomu Ronghua 31 |
| R049 | Shandong | Shouguang | Regular | Open field | 2023.07.22 | Xishi Caomei |
| R050 | Shandong | Shouguang | Regular | Open field | 2022.06.24 | Diana |
| R051 | Shandong | Shouguang | Regular | Open field | 2022.07.04 | Gaotang 100 |
| R052 | Shandong | Shouguang | Regular | Open field | 2022.06.17 | Fenruyi |
| R053 | Shandong | Shouguang | Regular | Open field | 2022.06.24 | Yali 1832 |
| R054 | Shandong | Shouguang | Regular | Open field | 2023.06.27 | Meicui No.1 |
| R055 | Shandong | Shouguang | Regular | Open field | 2022.07.04 | Yulinglong |
| R056 | Shandong | Shouguang | Regular | Open field | 2022.06.17 | Demei |
| R057 | Shandong | Shouguang | Regular | Open field | 2022.06.24 | Jiatelin |
| R058 | Shandong | Shouguang | Regular | Open field | 2022.06.17 | Haoshi Fanqie |
| R059 | Shandong | Shouguang | Regular | Open field | 2022.06.24 | Shengxia K3 |
| R060 | Shandong | Shouguang | Regular | Open field | 2022.06.24 | Meicui No.2 |
| R061 | Shandong | Shouguang | Regular | Open field | 2023.07.01 | Ailian 8 |
| R062 | Shandong | Shouguang | Regular | Open field | 2023.07.22 | Gushuo Shouhong |
| R063 | Shandong | Shouguang | Regular | Open field | 2023.07.01 | Gushuo Yipin |

| | | | | | | |
|---|---|---|---|---|---|---|
| R064 | Shandon g | Shouguang | Regula r | Open field | 2023.07.22 | Hongbaoshi Gaotang |
| R065 | Shandon g | Shouguang | Regula r | Open field | 2022.07.04 | Yuanwei No.1 |
| R066 | Shandon g | Shouguang | Regula r | Open field | 2023.06.27 | Yuanwei No.2 |
| C001 | Beijing | Changping | Cherry | Protected facility | 2022.06.17 | Unknown (B8271) |
| C002 | Beijing | Changping | Cherry | Protected facility | 2022.07.04 | B8272 |
| C003 | Beijing | Changping | Cherry | Protected facility | 2023.06.27 | B8273 |
| C004 | Beijing | Changping | Cherry | Protected facility | 2023.06.27 | B8274 |
| C005 | Beijing | Changping | Cherry | Protected facility | 2022.07.04 | B8454 |
| C006 | Beijing | Changping | Cherry | Protected facility | 2023.07.01 | B8455 |
| C007 | Beijing | Changping | Cherry | Protected facility | 2022.06.24 | B8456 |
| C008 | Beijing | Changping | Cherry | Protected facility | 2022.07.04 | B8457 |
| C009 | Beijing | Changping | Cherry | Protected facility | 2023.07.01 | B8458 |
| C010 | Beijing | Changping | Cherry | Protected facility | 2022.07.04 | B8459 |
| C011 | Beijing | Changping | Cherry | Protected facility | 2023.07.01 | B8460 |
| C012 | Beijing | Changping | Cherry | Protected facility | 2023.06.27 | B8461 |
| C013 | Beijing | Changping | Cherry | Protected facility | 2022.07.04 | B8462 |
| C014 | Beijing | Changping | Cherry | Protected facility | 2022.06.17 | B8463 |
| C015 | Beijing | Changping | Cherry | Protected facility | 2023.06.27 | B8464 |
| C016 | Beijing | Changping | Cherry | Protected facility | 2022.06.17 | B8465 |
| C017 | Beijing | Changping | Cherry | Protected facility | 2023.07.01 | B8466 |
| C018 | Beijing | Changping | Cherry | Protected facility | 2023.07.01 | B8467 |
| C019 | Beijing | Changping | Cherry | Protected facility | 2022.07.04 | B8468 |

| | | | | | | |
|---|---|---|---|---|---|---|
| C020 | Beijing | Changping | Cherry | Protected facility | 2023.07.01 | B8469 |
| C021 | Beijing | Changping | Cherry | Protected facility | 2022.07.04 | B8470 |
| C022 | Beijing | Changping | Cherry | Protected facility | 2022.07.04 | B8471 |
| C023 | Beijing | Changping | Cherry | Protected facility | 2023.06.27 | B8472 |
| C024 | Beijing | Changping | Cherry | Protected facility | 2023.06.27 | B8473 |
| C025 | Beijing | Changping | Cherry | Protected facility | 2022.06.17 | B8474 |
| C026 | Beijing | Changping | Cherry | Protected facility | 2022.07.04 | CHT3412Y |
| C027 | Beijing | Changping | Cherry | Protected facility | 2022.06.24 | CHT352Y |
| C028 | Beijing | Changping | Cherry | Protected facility | 2023.06.27 | Fanyu 1 |
| C029 | Beijing | Changping | Cherry | Protected facility | 2023.07.01 | Hei Xiaoge |
| C030 | Beijing | Changping | Cherry | Protected facility | 2022.06.17 | Huying 10 |
| C031 | Beijing | Changping | Cherry | Protected facility | 2022.06.17 | Laitaihong 1 |
| C032 | Beijing | Changping | Cherry | Protected facility | 2023.07.22 | Nandou |
| C033 | Beijing | Changping | Cherry | Protected facility | 2023.06.27 | Ningyinghong 3 |
| C034 | Beijing | Changping | Cherry | Protected facility | 2022.06.24 | Weima Hongzhu 1 |
| C035 | Beijing | Changping | Cherry | Protected facility | 2023.07.01 | Xindi |
| C036 | Beijing | Changping | Cherry | Protected facility | 2023.07.01 | Xinfei |
| C037 | Beijing | Changping | Cherry | Protected facility | 2023.07.22 | All-flesh Fruit |
| C038 | Beijing | Changping | Cherry | Protected facility | 2022.06.24 | All-flesh × H1706 |
| C039 | Beijing | Changping | Cherry | Protected facility | 2022.06.17 | H1706 |
| C040 | Shandong | Shouguang | Cherry | Open field | 2022.06.17 | Aiji Hongmei |
| C041 | Shandong | Shouguang | Cherry | Open field | 2022.06.24 | Lushou Hongniu |

| | | | | | | |
|---|---|---|---|---|---|---|
| C042 | Shandong | Shouguang | Cherry | Open field | 2023.07.22 | Lushou Chaoying |
| C043 | Shandong | Shouguang | Cherry | Open field | 2022.06.24 | Lushou Huadenglong |
| C044 | Shandong | Shouguang | Cherry | Open field | 2022.06.24 | Zijinxiang No.1 |
| C045 | Shandong | Shouguang | Cherry | Open field | 2023.07.22 | 21T30-1 |
| C046 | Shandong | Shouguang | Cherry | Open field | 2022.06.24 | 21T34-1 |
| C047 | Shandong | Shouguang | Cherry | Open field | 2023.07.01 | Fenjiali No.1 |
| C048 | Shandong | Shouguang | Cherry | Open field | 2023.07.22 | Fentao |
| C049 | Shandong | Shouguang | Cherry | Open field | 2022.07.04 | 21T35-2 |
| C050 | Shandong | Shouguang | Cherry | Open field | 2023.06.27 | 21T30-2 |
| C051 | Shandong | Shouguang | Cherry | Open field | 2022.06.24 | Shiji Xiaohong No.1 |
| C052 | Shandong | Shouguang | Cherry | Open field | 2022.06.17 | NC-60 |
| C053 | Shandong | Shouguang | Cherry | Open field | 2022.06.17 | Chixiazhu |
| C054 | Shandong | Shouguang | Cherry | Open field | 2022.06.17 | Hongzhenzhu |
| C055 | Shandong | Shouguang | Cherry | Open field | 2022.06.24 | Xidayinghong 1 |
| C056 | Shandong | Shouguang | Cherry | Open field | 2023.07.22 | Fushan 88 |
| C057 | Shandong | Shouguang | Cherry | Open field | 2022.06.24 | Qiande Xiaolongnü |
| C058 | Shandong | Shouguang | Cherry | Open field | 2023.07.01 | Huying K14 |
| C059 | Shandong | Shouguang | Cherry | Open field | 2022.06.24 | Qiande Xiaohong |
| C060 | Shandong | Shouguang | Cherry | Open field | 2023.07.22 | Ruibeika |
| C061 | Shandong | Shouguang | Cherry | Open field | 2022.06.24 | Monika |
| C062 | Shandong | Shouguang | Cherry | Open field | 2023.07.01 | Huying K16 |

| | | | | | | |
|---|---|---|---|---|---|---|
| C063 | Shandong | Shouguang | Cherry | Open field | 2023.07.22 | Hongxiaofan 035 |
| C064 | Shandong | Shouguang | Cherry | Open field | 2022.06.24 | Huying K10 |
| C065 | Shandong | Shouguang | Cherry | Open field | 2023.06.27 | Zijinxiang No.2 |
| C066 | Shandong | Shouguang | Cherry | Open field | 2022.06.17 | CT21-235 |
| C067 | Shandong | Shouguang | Cherry | Open field | 2022.07.04 | K2020-17 |
| C068 | Shandong | Shouguang | Cherry | Open field | 2022.06.24 | Fenjiali No.2 |
| C069 | Shandong | Shouguang | Cherry | Open field | 2023.07.22 | Qiande Ziwa |
| C070 | Shandong | Shouguang | Cherry | Open field | 2023.06.27 | Qiande Hongwa |
| C071 | Shandong | Shouguang | Cherry | Open field | 2022.07.04 | 21T30-1 (Rep) |
| C072 | Shandong | Shouguang | Cherry | Open field | 2022.06.17 | Huying K15 |
| C073 | Shandong | Shouguang | Cherry | Open field | 2023.07.01 | Huying K18 |
| C074 | Shandong | Shouguang | Cherry | Open field | 2023.07.22 | Xidayinghong 2 |
| C075 | Shandong | Shouguang | Cherry | Open field | 2023.06.27 | Yuhang 7 |
| C076 | Shandong | Shouguang | Cherry | Open field | 2022.07.04 | Haigena |
| C077 | Shandong | Shouguang | Cherry | Open field | 2022.06.17 | Huying K11 |
| C078 | Shandong | Shouguang | Cherry | Open field | 2023.07.22 | 21T34-1 (Rep) |
| C079 | Shandong | Shouguang | Cherry | Open field | 2023.07.22 | Huying K50 |
| C080 | Shandong | Shouguang | Cherry | Open field | 2023.07.01 | Shenghuang Xiaofanqie |
| C081 | Shandong | Shouguang | Cherry | Open field | 2022.06.17 | K2021-9 |
| C082 | Shandong | Shouguang | Cherry | Open field | 2022.06.24 | Xidayingfen 4 |
| C083 | Shandong | Shouguang | Cherry | Open field | 2022.06.24 | Fenxian |
| C084 | Shandong | Shouguang | Cherry | Open field | 2023.06.27 | K2020-26 |

| | | | | | | |
|---|---|---|---|---|---|---|
| C085 | Shandong | Shouguang | Cherry | Open field | 2023.06.27 | Misitila |
| C086 | Shandong | Shouguang | Cherry | Open field | 2023.07.01 | Yuhang 10 |
| C087 | Shandong | Shouguang | Cherry | Open field | 2023.07.01 | Ouxiu Hongying 1 |
| C088 | Shandong | Shouguang | Cherry | Open field | 2022.06.24 | Xidayingfen 1 |
| C089 | Shandong | Shouguang | Cherry | Open field | 2022.06.24 | Jianqiang 1 |
| C090 | Shandong | Shouguang | Cherry | Open field | 2022.06.17 | Hongtianmi |
| C091 | Shandong | Shouguang | Cherry | Open field | 2022.07.04 | Fenbaoshi |
| C092 | Shandong | Shouguang | Cherry | Open field | 2023.07.01 | Beibei |
| C093 | Shandong | Shouguang | Cherry | Open field | 2023.06.27 | Xidayingfen 2 |
| C094 | Shandong | Shouguang | Cherry | Open field | 2022.06.17 | Gushuo Fushan 88 |
| C095 | Shandong | Shouguang | Cherry | Open field | 2022.07.04 | Gushuo Huihuang |
| C096 | Shandong | Shouguang | Cherry | Open field | 2022.06.17 | Jinfenghuang |
| C097 | Shandong | Shouguang | Cherry | Open field | 2023.07.01 | Liangbei |
| C098 | Shandong | Shouguang | Cherry | Open field | 2023.07.22 | Shiji Xiaohong No.2 |
| C099 | Shandong | Shouguang | Cherry | Open field | 2023.07.01 | Shouerhong |

**Table S2.** Principal Component Analysis (PCA) Loading Matrices for Beijing regular Tomato.

| Beijing regular | PC1 | PC2 | PC3 |
|---|---|---|---|
| Lycopene | -0.28905 | 0.39695 | 0.43932 |
| Dry Matter | 0.41105 | 0.12576 | 0.12618 |
| Vc | 0.1639 | 0.43917 | -0.74087 |
| Total Acid | 0.38637 | -0.0895 | 0.27893 |
| Soluble Sugar | 0.35892 | 0.29444 | -0.12637 |
| Soluble Solids | 0.40786 | 0.14467 | 0.15284 |
| Protein | 0.31041 | -0.23558 | 0.10882 |
| β-Carotene | -0.16956 | 0.66907 | 0.2614 |
| Total 16 Amino Acids | 0.38867 | 0.12231 | 0.21185 |

**Table S3.** Principal Component Analysis (PCA) Loading Matrices for Beijing cherry Tomato.

| Beijing cherry | PC1 | PC2 | PC3 |
|---|---|---|---|
| Lycopene | -0.21625 | 0.18783 | 0.84968 |
| Dry Matter | 0.37888 | -0.00286 | -0.04593 |
| Vc | 0.36097 | 0.01185 | -0.14931 |
| Total Acid | 0.34026 | -0.17559 | 0.30696 |
| Soluble Sugar | 0.36345 | 0.02162 | -0.10665 |
| Soluble Solids | 0.37673 | -0.02143 | 0.01291 |
| Protein | 0.37094 | -0.01843 | 0.24448 |
| β-Carotene | 0.14124 | 0.96172 | -0.07485 |
| Total 16 Amino Acids | 0.36339 | -0.08698 | 0.28724 |

**Table S4.** Principal Component Analysis (PCA) Loading Matrices for Shandong regular Tomato.

| Shandong regular | PC1 | PC2 | PC3 |
|---|---|---|---|
| Lycopene | 0.26566 | 0.14504 | -0.43044 |
| Dry Matter | 0.45828 | -0.17625 | 0.3232 |
| Vc | -0.12261 | 0.1616 | 0.65709 |
| Total Acid | 0.4116 | 0.20473 | 0.18655 |
| Soluble Sugar | 0.36527 | -0.42865 | -0.25997 |
| Soluble Solids | 0.46687 | -0.15314 | 0.30253 |
| Protein | 0.13427 | 0.58792 | -0.21772 |
| β-Carotene | 0.30177 | -0.0901 | -0.18926 |
| Total 16 Amino Acids | 0.27289 | 0.56472 | 0.03693 |

**Table S5.** Principal Component Analysis (PCA) Loading Matrices for Shandong Cherry Tomatoes.

| Shandong Cherry | PC1 | PC2 | PC3 |
|---|---|---|---|
| Lycopene | 0.08838 | 0.25623 | 0.51847 |
| Dry Matter | 0.46657 | 0.01184 | -0.32068 |
| Vc | -0.226 | 0.43318 | -0.32643 |
| Total Acid | 0.38205 | 0.23609 | -0.12401 |
| Soluble Sugar | 0.45546 | -0.28823 | -0.00641 |
| Soluble Solids | 0.4636 | -0.01586 | -0.32476 |
| Protein | 0.04488 | 0.60516 | -0.00748 |
| β-Carotene | 0.29226 | -0.11389 | 0.5925 |
| Total 16 Amino Acids | 0.26012 | 0.47781 | 0.22316 |

**Table S6.** Pearson Correlation Coefficients and FDR-Adjusted p-Values for Quality Parameters: Beijing Regular Tomato (n=44)

| Pearson correlation coefficient | Lycopene | Dry Matter | Vc | Total Acid | Soluble Sugar | Soluble Solids | Protein | β-Carotene | Total 16 Amino Acids |
|---|---|---|---|---|---|---|---|---|---|
| Lycopene | 1 | -0.51687 | -0.21497 | -0.54266 | -0.44184 | -0.50422 | -0.50152 | 0.64624 | -0.41292 |
| Dry Matter | -0.51687 | 1 | 0.32242 | 0.83481 | 0.8442 | 0.98443 | 0.54931 | -0.25192 | 0.85224 |
| Vc | -0.21497 | 0.32242 | 1 | 0.13993 | 0.48578 | 0.29966 | 0.16046 | 0.10986 | 0.33646 |
| Total Acid | -0.54266 | 0.83481 | 0.13993 | 1 | 0.60922 | 0.82501 | 0.65484 | -0.35839 | 0.83662 |
| Soluble Sugar | -0.44184 | 0.8442 | 0.48578 | 0.60922 | 1 | 0.86775 | 0.38063 | -0.10759 | 0.69153 |
| Soluble Solids | -0.50422 | 0.98443 | 0.29966 | 0.82501 | 0.86775 | 1 | 0.54794 | -0.2123 | 0.83423 |

| | | | | | | | | | |
|---|---|---|---|---|---|---|---|---|---|
| Protein | -0.50152 | 0.54931 | 0.16046 | 0.65484 | 0.38063 | 0.54794 | 1 | -0.39772 | 0.67387 |
| β-Carotene | 0.64624 | -0.25192 | 0.10986 | -0.35839 | -0.10759 | -0.2123 | -0.39772 | 1 | -0.18586 |
| Total 16 Amino Acids | -0.41292 | 0.85224 | 0.33646 | 0.83662 | 0.69153 | 0.83423 | 0.67387 | -0.18586 | 1 |

| *P*-value | Lycopene | Dry Matter | Vc | Total Acid | Soluble Sugar | Soluble Solids | Protein | β-Carotene | Total 16 Amino Acids |
|---|---|---|---|---|---|---|---|---|---|
| Lycopene | -- | 3.28E-04 | 0.16111 | 1.41E-04 | 0.00268 | 4.83E-04 | 5.24E-04 | 2.16E-06 | 0.00534 |
| Dry Matter | 3.28E-04 | -- | 0.0328 | 1.88E-12 | 6.07E-13 | 2.40E-33 | 1.13E-04 | 0.09901 | 2.17E-13 |
| Vc | 0.16111 | 0.0328 | -- | 0.36497 | 8.29E-04 | 0.04813 | 0.2981 | 0.47775 | 0.02554 |
| Total Acid | 1.41E-04 | 1.88E-12 | 0.36497 | -- | 1.14E-05 | 5.71E-12 | 1.42E-06 | 0.0169 | 1.52E-12 |
| Soluble Sugar | 0.00268 | 6.07E-13 | 8.29E-04 | 1.14E-05 | -- | 2.47E-14 | 0.01081 | 0.48696 | 2.02E-07 |
| Soluble Solids | 4.83E-04 | 2.40E-33 | 0.04813 | 5.71E-12 | 2.47E-14 | -- | 1.18E-04 | 0.16651 | 2.01E-12 |
| Protein | 5.24E-04 | 1.13E-04 | 0.2981 | 1.42E-06 | 0.01081 | 1.18E-04 | -- | 0.00751 | 5.34E-07 |
| β-Carotene | 2.16E-06 | 0.09901 | 0.47775 | 0.0169 | 0.48696 | 0.16651 | 0.00751 | -- | 0.22707 |
| Total 16 Amino Acids | 0.00534 | 2.17E-13 | 0.02554 | 1.52E-12 | 2.02E-07 | 2.01E-12 | 5.34E-07 | 0.22707 | -- |

| FDR (Benjamini-Hochberg) | Lycopene | Dry Matter | Vc | Total Acid | Soluble Sugar | Soluble Solids | Protein | β-Carotene | Total 16 Amino Acids |
|---|---|---|---|---|---|---|---|---|---|
| Lycopene | -- | 0.0059 | 0.48333 | 0.00254 | 0.02412 | 0.00869 | 0.00943 | $2.59\times10^{-5}$ | 0.04806 |

| | | | | | | | | | |
|---|---|---|---|---|---|---|---|---|---|
| Dry Matter | 0.0059 | -- | 0.0984 | $5.65\times10^{-11}$ | $1.82\times10^{-11}$ | $2.88\times10^{-32}$ | 0.00203 | 0.29703 | $6.52\times10^{-12}$ |
| Vc | 0.48333 | 0.0984 | -- | 0.54746 | 0.00746 | 0.14439 | 0.44715 | 0.71663 | 0.07662 |
| Total Acid | 0.00254 | $5.65\times10^{-11}$ | 0.54746 | -- | 0.1026 | $1.71\times10^{-10}$ | $4.26\times10^{-5}$ | 0.0507 | $4.56\times10^{-11}$ |
| Soluble Sugar | 0.02412 | $1.82\times10^{-11}$ | 0.00746 | 0.1026 | -- | $7.41\times10^{-13}$ | 0.06486 | 0.73044 | $6.06\times10^{-6}$ |
| Soluble Solids | 0.00869 | $2.88\times10^{-32}$ | 0.14439 | $1.71\times10^{-10}$ | $7.41\times10^{-13}$ | -- | 0.00354 | 0.49953 | $6.03\times10^{-11}$ |
| Protein | 0.00943 | 0.00203 | 0.44715 | $4.26\times10^{-5}$ | 0.06486 | 0.00354 | -- | 0.02253 | $1.60\times10^{-5}$ |
| β-Carotene | $2.59\times10^{-5}$ | 0.29703 | 0.71663 | 0.0507 | 0.73044 | 0.49953 | 0.02253 | -- | 0.68121 |
| Total 16 Amino Acids | 0.04806 | $6.52\times10^{-12}$ | 0.07662 | $4.56\times10^{-11}$ | $6.06\times10^{-6}$ | $6.03\times10^{-11}$ | $1.60\times10^{-5}$ | 0.68121 | -- |

**Table S7.** Pearson Correlation Coefficients and FDR-Adjusted p-Values for Quality Parameters: Beijing Cherry Tomato (n=39)

| Pearson correlation coefficient | Lycopene | Dry Matter | Vc | Total Acid | Soluble Sugar | Soluble Solids | Protein | β-Carotene | Total 16 Amino Acids |
|---|---|---|---|---|---|---|---|---|---|
| Lycopene | 1 | -0.55759 | -0.57899 | -0.37229 | -0.55722 | -0.51282 | -0.39557 | -0.11849 | -0.36789 |
| Dry Matter | -0.55759 | 1 | 0.94372 | 0.81806 | 0.94123 | 0.98485 | 0.92359 | 0.34696 | 0.90215 |
| Vc | -0.57899 | 0.94372 | 1 | 0.73549 | 0.8962 | 0.92596 | 0.8506 | 0.34018 | 0.82088 |
| Total Acid | -0.37229 | 0.81806 | 0.73549 | 1 | 0.74711 | 0.83255 | 0.93171 | 0.18497 | 0.9298 |
| Soluble Sugar | -0.55722 | 0.94123 | 0.8962 | 0.74711 | 1 | 0.93778 | 0.8607 | 0.34893 | 0.84491 |
| Soluble Solids | -0.51282 | 0.98485 | 0.92596 | 0.83255 | 0.93778 | 1 | 0.92728 | 0.32379 | 0.90183 |

| | | | | | | | | | |
|---|---|---|---|---|---|---|---|---|---|
| Protein | -0.39557 | 0.92359 | 0.8506 | 0.93171 | 0.8607 | 0.92728 | 1 | 0.33166 | 0.98544 |
| β-Carotene | -0.11849 | 0.34696 | 0.34018 | 0.18497 | 0.34893 | 0.32379 | 0.33166 | 1 | 0.26169 |
| Total 16 Amino Acids | -0.36789 | 0.90215 | 0.82088 | 0.9298 | 0.84491 | 0.90183 | 0.98544 | 0.26169 | 1 |

| *P*-value | Lycopene | Dry Matter | Vc | Total Acid | Soluble Sugar | Soluble Solids | Protein | β-Carotene | Total 16 Amino Acids |
|---|---|---|---|---|---|---|---|---|---|
| Lycopene | -- | 2.26E-04 | 1.13E-04 | 0.0196 | 2.29E-04 | 8.44E-04 | 0.01268 | 0.47249 | 0.02121 |
| Dry Matter | 2.26E-04 | -- | 2.29E-19 | 2.03E-10 | 5.00E-19 | 9.30E-30 | 5.52E-17 | 0.03047 | 4.45E-15 |
| Vc | 1.13E-04 | 2.29E-19 | -- | 9.64E-08 | 1.26E-14 | 3.14E-17 | 7.11E-12 | 0.03409 | 1.56E-10 |
| Total Acid | 0.0196 | 2.03E-10 | 9.64E-08 | -- | 4.68E-08 | 4.99E-11 | 7.40E-18 | 0.25961 | 1.21E-17 |
| Soluble Sugar | 2.29E-04 | 5.00E-19 | 1.26E-14 | 4.68E-08 | -- | 1.40E-18 | 2.13E-12 | 0.02947 | 1.35E-11 |
| Soluble Solids | 8.44E-04 | 9.30E-30 | 3.14E-17 | 4.99E-11 | 1.40E-18 | -- | 2.28E-17 | 0.04434 | 4.71E-15 |
| Protein | 0.01268 | 5.52E-17 | 7.11E-12 | 7.40E-18 | 2.13E-12 | 2.28E-17 | -- | 0.03915 | 4.48E-30 |
| β-Carotene | 0.47249 | 0.03047 | 0.03409 | 0.25961 | 0.02947 | 0.04434 | 0.03915 | -- | 0.10756 |
| Total 16 Amino Acids | 0.02121 | 4.45E-15 | 1.56E-10 | 1.21E-17 | 1.35E-11 | 4.71E-15 | 4.48E-30 | 0.10756 | -- |

| FDR （Benjamini-Hochberg ） | Lycopene | Dry Matter | Vc | Total Acid | Soluble Sugar | Soluble Solids | Protein | β-Carotene | Total 16 Amino Acids |
|---|---|---|---|---|---|---|---|---|---|

| | | | | | | | | | |
|---|---|---|---|---|---|---|---|---|---|
| Lycopene | -- | 0.00407 | 0.00203 | 0.07056 | 0.00412 | 0.01013 | 0.04563 | 0.47249 | 0.07636 |
| Dry Matter | 0.00407 | -- | $8.24\times10^{-18}$ | $7.30\times10^{-9}$ | $1.80\times10^{-17}$ | $3.35\times10^{-28}$ | $1.99\times10^{-15}$ | 0.54846 | $1.60\times10^{-13}$ |
| Vc | 0.00203 | $8.24\times10^{-18}$ | -- | $3.47\times10^{-6}$ | $4.54\times10^{-13}$ | $1.13\times10^{-15}$ | $2.56\times10^{-10}$ | 0.61362 | $5.62\times10^{-9}$ |
| Total Acid | 0.07056 | $7.30\times10^{-9}$ | $3.47\times10^{-6}$ | -- | $1.68\times10^{-6}$ | $1.80\times10^{-9}$ | $2.66\times10^{-16}$ | 1 | $4.36\times10^{-16}$ |
| Soluble Sugar | 0.00412 | $1.80\times10^{-17}$ | $4.54\times10^{-13}$ | $1.68\times10^{-6}$ | -- | $5.04\times10^{-17}$ | $7.67\times10^{-11}$ | 1 | $4.86\times10^{-10}$ |
| Soluble Solids | 0.01013 | $3.35\times10^{-28}$ | $1.13\times10^{-15}$ | $1.80\times10^{-9}$ | $5.04\times10^{-17}$ | -- | $8.21\times10^{-16}$ | 1 | $1.70\times10^{-13}$ |
| Protein | 0.04563 | $1.99\times10^{-15}$ | $2.56\times10^{-10}$ | $2.66\times10^{-16}$ | $7.67\times10^{-11}$ | $8.21\times10^{-16}$ | -- | 1 | $1.61\times10^{-28}$ |
| β-Carotene | 0.47249 | 0.54846 | 0.61362 | 1 | 1 | 1 | 1 | -- | 1 |
| Total 16 Amino Acids | 0.07636 | $1.60\times10^{-13}$ | $5.62\times10^{-9}$ | $4.36\times10^{-16}$ | $4.86\times10^{-10}$ | $1.70\times10^{-13}$ | $1.61\times10^{-28}$ | 1 | -- |

**Table S8.** Pearson Correlation Coefficients and FDR-Adjusted p-Values for Quality Parameters: handong Regular Tomato (n=22)

| Pearson correlation coefficient | Lycopene | Dry Matter | Vc | Total Acid | Soluble Sugar | Soluble Solids | Protein | β-Carotene | Total 16 Amino Acids |
|---|---|---|---|---|---|---|---|---|---|
| Lycopene | 1 | 0.13616 | -0.40019 | 0.24565 | 0.40794 | 0.18263 | 0.3857 | 0.09763 | 0.36533 |
| Dry Matter | 0.13616 | 1 | 0.09523 | 0.59008 | 0.58868 | 0.98727 | -0.1231 | 0.31584 | 0.22306 |
| Vc | -0.40019 | 0.09523 | 1 | 0.03708 | -0.59487 | 0.09522 | -0.11142 | -0.2927 | 0.12382 |
| Total Acid | 0.24565 | 0.59008 | 0.03708 | 1 | 0.22278 | 0.5807 | 0.29989 | 0.26024 | 0.62391 |
| Soluble Sugar | 0.40794 | 0.58868 | -0.59487 | 0.22278 | 1 | 0.59023 | -0.31394 | 0.49109 | -0.22435 |
| Soluble Solids | 0.18263 | 0.98727 | 0.09522 | 0.5807 | 0.59023 | 1 | -0.06888 | 0.32956 | 0.25348 |
| Protein | 0.3857 | -0.1231 | -0.11142 | 0.29989 | -0.31394 | -0.06888 | 1 | 0.14771 | 0.86628 |
| β-Carotene | 0.09763 | 0.31584 | -0.2927 | 0.26024 | 0.49109 | 0.32956 | 0.14771 | 1 | 0.16988 |
| Total 16 Amino Acids | 0.36533 | 0.22306 | 0.12382 | 0.62391 | -0.22435 | 0.25348 | 0.86628 | 0.16988 | 1 |

| *P*-value | Lycopene | Dry Matter | Vc | Total Acid | Soluble Sugar | Soluble Solids | Protein | β-Carotene | Total 16 Amino Acids |
|---|---|---|---|---|---|---|---|---|---|
| Lycopene | -- | 0.54572 | 0.06496 | 0.27049 | 0.05947 | 0.41593 | 0.07626 | 0.66557 | 0.09455 |
| Dry Matter | 0.54572 | -- | 0.67336 | 0.00384 | 0.00395 | 1.92E-17 | 0.58522 | 0.15217 | 0.31836 |

| | | | | | | | | | |
|---|---|---|---|---|---|---|---|---|---|
| Vc | 0.06496 | 0.67336 | -- | 0.86986 | 0.0035 | 0.67338 | 0.62157 | 0.1862 | 0.583 |
| Total Acid | 0.27049 | 0.00384 | 0.86986 | -- | 0.319 | 0.0046 | 0.17512 | 0.24214 | 0.00192 |
| Soluble Sugar | 0.05947 | 0.00395 | 0.0035 | 0.319 | -- | 0.00383 | 0.15478 | 0.02029 | 0.3155 |
| Soluble Solids | 0.41593 | 1.92E-17 | 0.67338 | 0.0046 | 0.00383 | -- | 0.76068 | 0.1342 | 0.25502 |
| Protein | 0.07626 | 0.58522 | 0.62157 | 0.17512 | 0.15478 | 0.76068 | -- | 0.51183 | 1.88E-07 |
| β-Carotene | 0.66557 | 0.15217 | 0.1862 | 0.24214 | 0.02029 | 0.1342 | 0.51183 | -- | 0.44976 |
| Total 16 Amino Acids | 0.09455 | 0.31836 | 0.583 | 0.00192 | 0.3155 | 0.25502 | 1.88E-07 | 0.44976 | -- |

| FDR (Benjamini-Hochberg) | Lycopene | Dry Matter | Vc | Total Acid | Soluble Sugar | Soluble Solids | Protein | β-Carotene | Total 16 Amino Acids |
|---|---|---|---|---|---|---|---|---|---|
| Lycopene | -- | 0.9823 | 0.19488 | 0.48688 | 0.17841 | 0.74927 | 0.22878 | 1 | 0.28365 |
| Dry Matter | 0.9823 | -- | 1 | 0.04608 | 0.0474 | $6.91\times10^{-16}$ | 1 | 0.91302 | 1 |
| Vc | 0.19488 | 1 | -- | 1 | 0.042 | 1 | 1 | 0.5586 | 1 |
| Total Acid | 0.48688 | 0.04608 | 1 | -- | 0.5742 | 0.0552 | 0.52536 | 0.72642 | 0.02304 |
| Soluble Sugar | 0.17841 | 0.0474 | 0.042 | 0.5742 | -- | 0.04596 | 0.46434 | 0.06087 | 0.5742 |
| Soluble Solids | 0.74927 | $6.91\times10^{-16}$ | 1 | 0.0552 | 0.04596 | -- | 1 | 0.4026 | 0.76506 |
| Protein | 0.22878 | 1 | 1 | 0.52536 | 0.46434 | 1 | -- | 1 | $2.26\times10^{-6}$ |
| β-Carotene | 1 | 0.91302 | 0.5586 | 0.72642 | 0.06087 | 0.4026 | 1 | -- | 1 |
| Total 16 Amino Acids | 0.28365 | 1 | 1 | 0.02304 | 0.5742 | 0.76506 | $2.26\times10^{-6}$ | 1 | -- |

**Table S9.** Pearson Correlation Coefficients and FDR-Adjusted p-Values for Quality Parameters: Shandong Cherry Tomato (n=60)

| Pearson correlation coefficient | Lycopene | Dry Matter | Vc | Total Acid | Soluble Sugar | Soluble Solids | Protein | β-Carotene | Total 16 Amino Acids |
|---|---|---|---|---|---|---|---|---|---|
| Lycopene | 1 | 0.0883 | 0.08687 | 0.13832 | 0.02383 | 0.02279 | 0.23467 | 0.15442 | 0.34716 |
| Dry Matter | 0.0883 | 1 | -0.2081 | 0.6269 | 0.79639 | 0.96981 | 0.07204 | 0.25934 | 0.34899 |
| Vc | 0.08687 | -0.2081 | 1 | -0.12124 | -0.66219 | -0.21454 | 0.48597 | -0.47546 | 0.13701 |
| Total Acid | 0.13832 | 0.6269 | -0.12124 | 1 | 0.47055 | 0.57871 | 0.40548 | 0.2372 | 0.54967 |
| Soluble Sugar | 0.02383 | 0.79639 | -0.66219 | 0.47055 | 1 | 0.80568 | -0.3567 | 0.53852 | 0.10786 |
| Soluble Solids | 0.02279 | 0.96981 | -0.21454 | 0.57871 | 0.80568 | 1 | 0.0332 | 0.29772 | 0.34132 |
| Protein | 0.23467 | 0.07204 | 0.48597 | 0.40548 | -0.3567 | 0.0332 | 1 | -0.08369 | 0.74921 |
| β-Carotene | 0.15442 | 0.25934 | -0.47546 | 0.2372 | 0.53852 | 0.29772 | -0.08369 | 1 | 0.33567 |
| Total 16 Amino Acids | 0.34716 | 0.34899 | 0.13701 | 0.54967 | 0.10786 | 0.34132 | 0.74921 | 0.33567 | 1 |

| *P*-value | Lycopene | Dry Matter | Vc | Total Acid | Soluble Sugar | Soluble Solids | Protein | β-Carotene | Total 16 Amino Acids |
|---|---|---|---|---|---|---|---|---|---|
| Lycopene | -- | 0.50229 | 0.50924 | 0.29192 | 0.85655 | 0.86277 | 0.07111 | 0.23878 | 0.00658 |

| | | | | | | | | | |
|---|---|---|---|---|---|---|---|---|---|
| Dry Matter | 0.50229 | -- | 0.11059 | 8.38E-08 | 2.79E-14 | 3.05E-37 | 0.58439 | 0.0454 | 0.00628 |
| Vc | 0.50924 | 0.11059 | -- | 0.35612 | 8.31E-09 | 0.09974 | 8.27E-05 | 1.24E-04 | 0.29653 |
| Total Acid | 0.29192 | 8.38E-08 | 0.35612 | -- | 1.48E-04 | 1.28E-06 | 0.00131 | 0.06803 | 5.40E-06 |
| Soluble Sugar | 0.85655 | 2.79E-14 | 8.31E-09 | 1.48E-04 | -- | 8.27E-15 | 0.00515 | 9.06E-06 | 0.41205 |
| Soluble Solids | 0.86277 | 3.05E-37 | 0.09974 | 1.28E-06 | 8.27E-15 | -- | 0.80116 | 0.02087 | 0.00761 |
| Protein | 0.07111 | 0.58439 | 8.27E-05 | 0.00131 | 0.00515 | 0.80116 | -- | 0.52497 | 5.76E-12 |
| β-Carotene | 0.23878 | 0.0454 | 1.24E-04 | 0.06803 | 9.06E-06 | 0.02087 | 0.52497 | -- | 0.00874 |
| Total 16 Amino Acids | 0.00658 | 0.00628 | 0.29653 | 5.40E-06 | 0.41205 | 0.00761 | 5.76E-12 | 0.00874 | -- |

| FDR (Benjamini-Hochberg ) | Lycopene | Dry Matter | Vc | Total Acid | Soluble Sugar | Soluble Solids | Protein | β-Carotene | Total 16 Amino Acids |
|---|---|---|---|---|---|---|---|---|---|
| Lycopene | -- | 0.75344 | 0.76386 | 0.43788 | 1 | 1 | 0.21333 | 0.71634 | 0.03948 |
| Dry Matter | 0.75344 | -- | 0.33277 | $1.51\times10^{-6}$ | $5.02\times10^{-13}$ | $1.10\times10^{-35}$ | 1 | 0.1362 | 0.03768 |
| Vc | 0.76386 | 0.33277 | -- | 0.53418 | $1.50\times10^{-7}$ | 0.29922 | 0.00149 | 0.00223 | 0.53418 |
| Total Acid | 0.43788 | $1.51\times10^{-6}$ | 0.53418 | -- | 0.00266 | $2.30\times10^{-5}$ | 0.02358 | 0.40818 | $9.72\times10^{-5}$ |
| Soluble Sugar | 1 | $5.02\times10^{-13}$ | $1.50\times10^{-7}$ | 0.00266 | -- | $1.49\times10^{-13}$ | 0.04635 | $8.15\times10^{-5}$ | 1 |
| Soluble Solids | 1 | $1.10\times10^{-35}$ | 0.29922 | $2.30\times10^{-5}$ | $1.49\times10^{-13}$ | -- | 1 | 0.18783 | 0.06849 |
| Protein | 0.21333 | 1 | 0.00149 | 0.02358 | 0.04635 | 1 | -- | 1 | $1.04\times10^{-10}$ |
| β-Carotene | 0.71634 | 0.1362 | 0.00223 | 0.40818 | $8.15\times10^{-5}$ | 0.18783 | 1 | -- | 0.07866 |

| | | | | | | | | | |
|---|---|---|---|---|---|---|---|---|---|
| Total 16 Amino Acids | 0.03948 | 0.03768 | 0.53418 | 9.72×10⁻⁵ | 1 | 0.06849 | 1.04×10⁻¹⁰ | 0.07866 | -- |
